# Advances in Noninvasive Molecular Imaging Probes for Liver Fibrosis Diagnosis

**DOI:** 10.34133/bmr.0042

**Published:** 2024-07-01

**Authors:** Shaofang Chen, Danping Zhuang, Qingyun Jia, Bing Guo, Genwen Hu

**Affiliations:** ^1^ Department of Radiology, Shenzhen People’s Hospital (The Second Clinical Medical College, Jinan University; The First Affiliated Hospital, Southern University of Science and Technology), Shenzhen 518020, Guangdong, China.; ^2^ School of Science, Shenzhen Key Laboratory of Flexible Printed Electronics Technology, Shenzhen Key Laboratory of Advanced Functional Carbon Materials Research and Comprehensive Application, Harbin Institute of Technology, Shenzhen 518055, China.

## Abstract

Liver fibrosis is a wound-healing response to chronic liver injury, which may lead to cirrhosis and cancer. Early-stage fibrosis is reversible, and it is difficult to precisely diagnose with conventional imaging modalities such as magnetic resonance imaging, positron emission tomography, single-photon emission computed tomography, and ultrasound imaging. In contrast, probe-assisted molecular imaging offers a promising noninvasive approach to visualize early fibrosis changes in vivo, thus facilitating early diagnosis and staging liver fibrosis, and even monitoring of the treatment response. Here, the most recent progress in molecular imaging technologies for liver fibrosis is updated. We start by illustrating pathogenesis for liver fibrosis, which includes capillarization of liver sinusoidal endothelial cells, cellular and molecular processes involved in inflammation and fibrogenesis, as well as processes of collagen synthesis, oxidation, and cross-linking. Furthermore, the biological targets used in molecular imaging of liver fibrosis are summarized, which are composed of receptors on hepatic stellate cells, macrophages, and even liver collagen. Notably, the focus is on insights into the advances in imaging modalities developed for liver fibrosis diagnosis and the update in the corresponding contrast agents. In addition, challenges and opportunities for future research and clinical translation of the molecular imaging modalities and the contrast agents are pointed out. We hope that this review would serve as a guide for scientists and students who are interested in liver fibrosis imaging and treatment, and as well expedite the translation of molecular imaging technologies from bench to bedside.

## Introduction

Liver fibrosis is a wound-healing response to chronic liver injury, manifested as the accumulation of extracellular matrix (ECM) proteins, resulting from a variety of causes, such as hepatitis B or C infections, nonalcoholic steatohepatitis (NASH), among others [1–3]. Patients undergoing fibrogenic progression are at high risk of developing cirrhosis and even primary liver cancer, especially hepatocellular carcinoma (HCC) [4–6]. Over the past few decades, liver disease has emerged as one of the major causes of death worldwide [7]. Global statistics estimate that liver disease accounts for approximately 2 million deaths annually, with cirrhosis and liver cancer causing 1.16 million and 788,000 deaths, respectively. They are the 11th and 16th most common causes of death each year [8].

Liver fibrosis, a common feature of nearly all chronic liver diseases [9], currently has no approved anti-fibrotic therapies by the Food and Drug Administration (FDA) for clinical application [10]. However, numerous pieces of evidence demonstrates that liver fibrosis can be reversed if the injury source is removed [11,12]. Therefore, reliable methods for early and staged diagnosis of liver fibrosis are essential to stop the progression of liver fibrosis to decompensated cirrhosis or HCC [13,14]. While liver biopsy is considered the gold standard for assessing liver fibrosis [15], it has multiple drawbacks. These include potential complications, low patient compliance with repeat tests, and significant sampling error [13,16]. As a result, to overcome the limitations of liver biopsy, there is a pressing need for noninvasive diagnostic methods for liver fibrosis, such as serum markers and imaging techniques [13,17]. Despite serum markers allowing for dynamic monitoring of liver fibrosis, they are susceptible to nonliver-specific factors such as inflammation, which can lead to less accurate results [17–19].

Current imaging techniques used to study liver fibrosis include ultrasound (US), computed tomography (CT), positron emission tomography (PET), single-photon emission CT (SPECT), magnetic resonance imaging (MRI), fluorescence imaging (FI), photoacoustic imaging (PAI), Raman imaging, and so on. US is the most commonly used imaging technique for clinical use to assess chronic liver disease, but it is not sensitive to the staging of liver fibrosis [20,21]. SPECT and PET are imaging methods that monitor metabolic function by measuring the distribution of radiolabeled tracers [22]. However, CT, SPECT, and PET are known to have ionizing radiation [23]. MRI offers high resolution and soft tissue contrast, enabling assessment of intermediate and advanced liver fibrosis stages, but has lower sensitivity in early-stage diagnosis [24]. To further improve the resolution and sensitivity of diagnosis, dynamic contrast-enhanced MRI (DCE-MRI) with gadolinium (Gd)-based contrast agents is often used, but it cannot be utilized in patients with renal failure [25,26]. Elastography reflects tissue stiffness, and an increase in stiffness is related to the severity of fibrosis. It can be categorized as US-based or magnetic resonance-based elastography (MRE). The most commonly assessed elastography method for liver stiffness is transient elastography (TE), which is US-based, but TE has lower diagnostic reliability in obese patients [27–29]. MRE is more accurate but requires expensive equipment and expertise [30,31]. FI and PAI have attracted extensive research attention due to their rapid feedback, high sensitivity, cost-effectiveness, and lack of ionizing radiation [32,33]. Raman imaging exhibits exceptional sensitivity, photostability, and almost no autofluorescence problems. However, the penetration depth of FI, PAI, and Raman imaging may be limited [34,35]. As mentioned above, while these imaging modalities are helpful in the evaluation and staging of hepatic fibrosis, particularly for mid- to late-stage liver fibrosis (F2-4), they are of lower sensitivity and precision in identifying and quantifying early-stage (F0-1) liver fibrosis [24]. Therefore, developing a noninvasive, precise, and repeatable assay for early detection, monitoring of disease progression, and assessment of response to liver fibrosis treatment would be highly desirable [6].

Molecular imaging applies conventional imaging techniques and introduces molecular probes to visualize, characterize, and quantify in vivo biological processes at the molecular and cellular levels, thus identifying the expression of molecular markers [36,37]. Indeed, liver fibrosis involves various pathophysiological processes. There are some critical targets in these processes, and molecular imaging typically utilizes specific molecular probes to engage with these targets, allowing for earlier disease detection by identifying a minimal number of abnormalities [36,37]. In essence, molecular imaging provides a more precise method for diagnosing liver fibrosis than conventional imaging techniques.

**Fig. 1.**
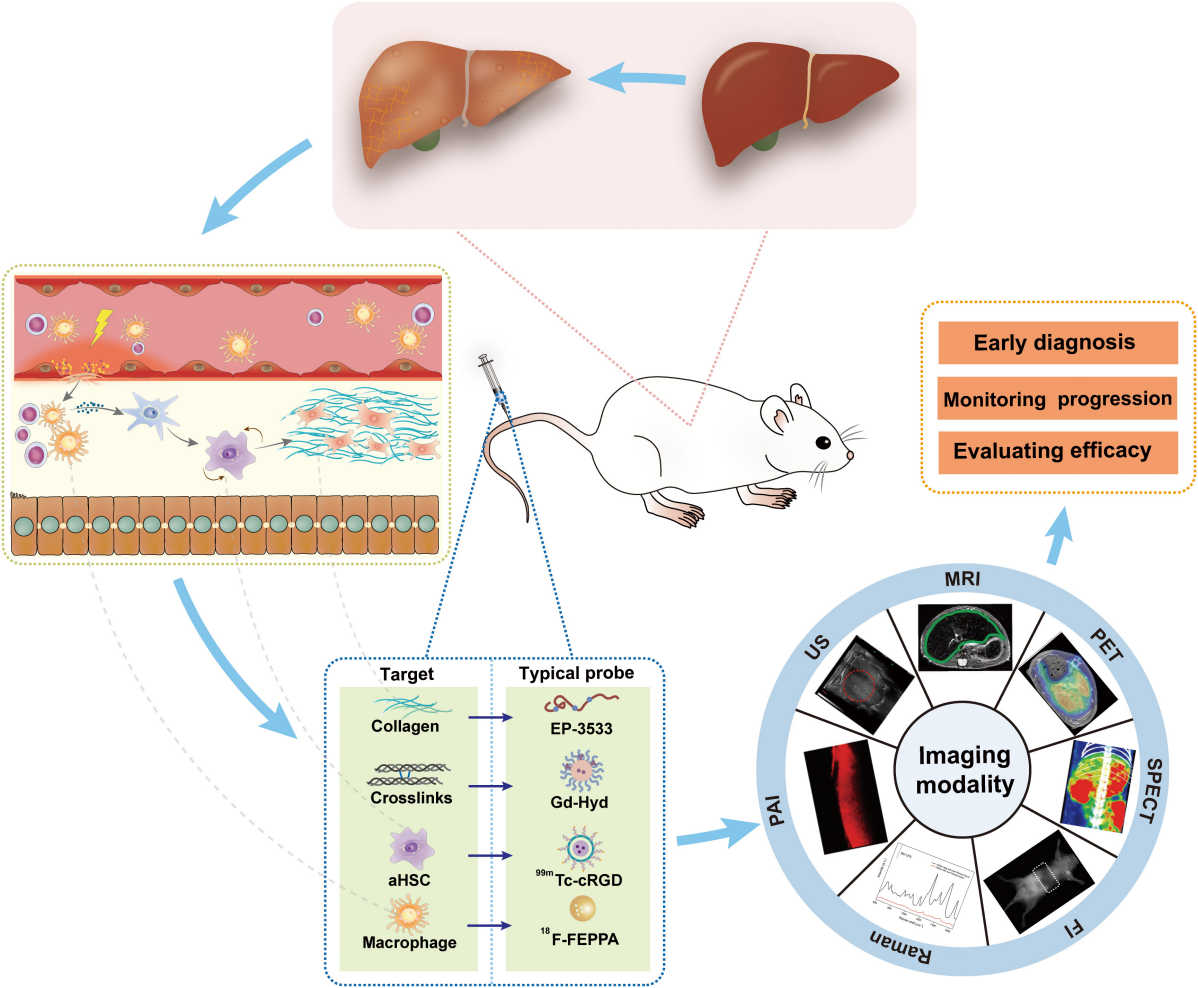
Schematic illustration of potential targets of liver fibrosis and molecular probes and imaging modalities used in liver fibrosis.

Noninvasive molecular imaging for early and staged diagnosis of liver fibrosis has garnered considerable interest, as reflected in recent reviews. For instance, Zhou et al. [6] summarized the MRI contrast agents available for chronic liver disease. Montesi et al. [38] and Désogère et al. [39] reviewed available molecular probes for assessing a variety of fibrotic diseases, including cardiac, pulmonary, and hepatic. Hagan et al. [40] described the application and advantages of US-based tests and MRI-based tests that have been examined and employed for liver fibrosis evaluation. Bai et al. [41] reviewed numerous inorganic or organic nanoparticles (NPs) for liver fibrosis theranostics, including liposomes, inorganic NPs, protein NPs, polymer NPs, and hybrid NPs. Baues et al. [42] summarized the noninvasive molecular imaging techniques for diagnosing and staging fibrosis diseases. Yu et al. [15] and Yip et al. [43] reviewed blood and imaging biomarkers of inflammation in nonalcoholic fatty liver disease (NAFLD), emphasizing the emerging role of artificial intelligence (AI) and machine learning in diagnostics. Notably, most previous reviews focused on the common mechanisms of various fibrotic diseases and summarized molecular probes from the perspective of pathological development. As currently the research on molecular imaging of liver fibrosis is growing rapidly, we herein offer a timely update on this topic with a special focus on how to achieve precise diagnosis and staging of liver fibrosis based on molecular imaging probes. At the beginning, a comprehensive introduction to the pathological mechanisms of liver fibrosis and potential targets for imaging diagnosis is presented, followed by the application of molecular probes from the perspective of multiple imaging modalities. Furthermore, the principles of different imaging modalities and the design strategies on molecular imaging probes are pointed out. It is expected that this review would serve as a guide for researchers who hold interest in development and application of molecular imaging probes on diagnosis and treatment of liver fibrosis in the future (Fig. 1).

## Pathogenesis of Liver Fibrosis

During the process of liver fibrosis, injury causes 3 overlapping but distinct stages: inflammation, ECM deposition, and granulation tissue formation, along with tissue remodeling and hardening [44]. These processes require activating and coordinating various intracellular and intercellular pathways to maintain tissue integrity and homeostasis. When ECM synthesis exceeds degradation, excessive matrix deposition may contribute to hepatic fibrosis, scar formation, and destruction of tissue structure and organ function. In addition, injury leads to the abnormal release of various cytokines that are involved in and regulate the development of liver fibrosis [45].

### LSEC capillarization

The liver comprises various types of cells classified as parenchymal cells (hepatocytes) and nonparenchymal cells, including liver sinusoidal endothelial cells (LSECs), hepatic stellate cells (HSCs), and macrophages [46]. The sinusoid is the microvascular unit of the liver, and its wall is composed of LSECs. LSECs are highly specialized endothelial cells with unique morphology and functions and are highly endocytic. LSECs contain many small fenestrations, about 100 to 150 nm in diameter [47]. These open fenestrations lack diaphragms and basal lamina and are organized into sieve plates, making LSECs highly permeable [48,49]. Moreover, the number and size of the fenestration can rapidly change depending on factors such as alcohol, dietary composition, and fasting, thus acting as a “dynamic filter” [50]. Up to one-third of these fenestrations, along with components such as microtubules, caveoli, and coated pits, form complex labyrinths that make up a transport network regulating the flow of substances in and out of the cell [51]. Thus, the primary function of LSECs is to reduce obstacles to the bidirectional transport of fluid and small or soluble substances between the blood and the Disse space while blocking the entry of larger circulating particles such as blood cells, platelets, and celiac particles [52].

**Fig. 2.**
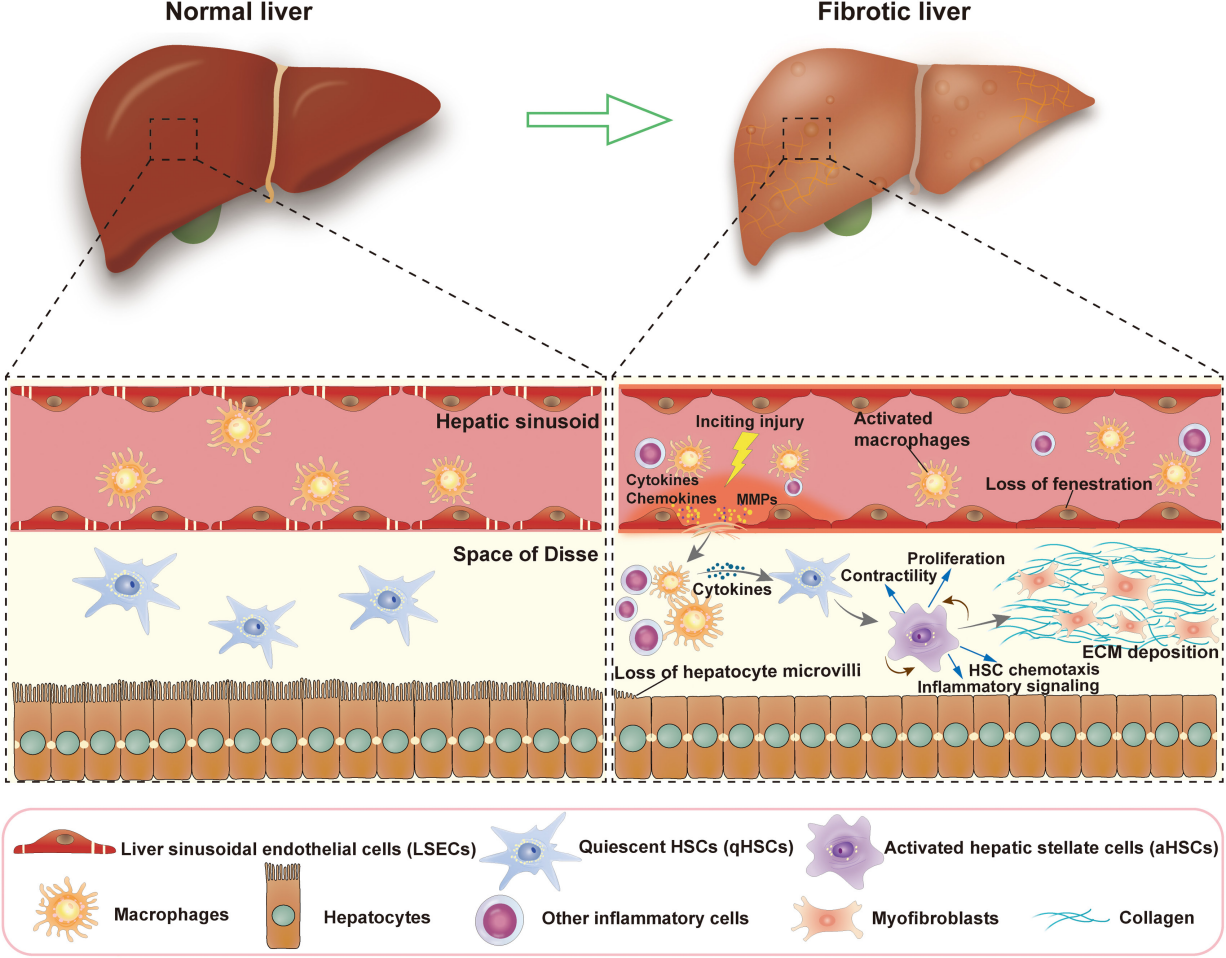
Schematic representation of capillarization of LSECs and cellular and molecular processes involved in liver fibrosis.

Under pathological conditions, LSECs play a critical part in the occurrence and development of liver fibrosis. The loss of LSEC fenestration and the formation of a subendothelial basement membrane lead to hepatic sinusoid resembling a capillary elsewhere in the body, a phenomenon known as LSEC capillarization (Fig. 2). The capillarization of LSECs reduces the exchange of material between blood and hepatocytes. Importantly, capillarization is an early event that precedes the onset of liver fibrosis [53,54] and maybe a permissive condition for the development of fibrosis [55,56]. Liver injury results in the recruitment of bone marrow endothelial progenitor cells that engraft but fail to differentiate into mature LSECs, leading to LSEC capillarization [57]. These dedifferentiated LSECs impair the activity of endothelial nitric oxide (NO) synthase, leading to decreased NO production. This results in vasoconstriction and increased intrahepatic resistance, which is accompanied by the development of fibrosis [58].

On the other hand, differentiated LSECs in a normal liver have a gatekeeper function to promote the quiescence of HSCs [59]. However, capillarized LSECs allow the activation of HSCs due to the inability to shed heparin-binding epidermal growth factor-like growth factors from the cytosolic membrane, which is an early event in liver fibrosis [57]. In summary, LSECs undergo capillarization and lose their protective properties in the liver, promoting angiogenesis, vasoconstriction, and the initiation and progression of liver fibrosis [54]. In fact, maintaining the differentiation of LSEC requires vascular endothelial growth factor (VEGF)-stimulated NO working through soluble guanylate cyclase (sGC) activation, as well as VEGF working through a NO-independent pathway. Xie et al. [55] conducted an in vitro experiment where they cultured activated HSCs (aHSCs) with dedifferentiated LSECs and/or an sGC activator. The results showed a significant reduction in the number of α-smooth muscle actin (α-SMA)-positive cells in HSCs cocultured with dedifferentiated LSECs and the sGC activator compared to aHSCs cultured alone or with the addition of the sGC activator. This suggests that although the sGC activator can reverse HSC activation in vitro, its effect is limited. However, coculturing with dedifferentiated LSECs and the sGC activator helps restore the normal phenotype of LSECs, and the differentiated LSECs cause aHSCs to return to a quiescent state, thereby preventing fibrosis progression. This demonstrated that restoring LSEC phenotype in the liver may be a feasible way to promote fibrosis regression or prevent disease progression.

### Cellular and molecular processes involved in tissue inflammation and fibrogenesis

Liver damage, often related to disease, initiates a complex series of cellular and molecular reactions that lead to tissue fibrosis. At the cellular level, various stimuli can damage resident epithelial cells, predominantly endothelial cells, leading to an increased release of inflammatory mediators such as cytokines and chemokines. Simultaneously, epithelial and endothelial cells generate matrix metalloproteinases (MMPs), including MMP-2 and MMP-9. The up-regulation of MMP-2 and MMP-9 may lead to the destruction of the basement membrane and remodeling of the ECM [60,61]. Additionally, MMP-9 may disrupt vascular integrity by breaking down cell adhesion molecules and serve as a critical mediator for leukocyte recruitment in liver injury [62,63]. These processes attract inflammatory cells, mainly macrophages, to the damaged area. After inflammatory cells enter the space of Disse, cytokines and chemokines are produced, which induce the activation of HSCs. Other matrix-producing cells such as marrow-derived cells and portal fibroblasts also participate [64,65]. These activated cells subsequently transdifferentiate into myofibroblasts, leading to increased expression and secretion of ECM, resulting in matrix deposition. This process drives the process of fibrogenesis [6,66] (Fig. 2).

Although multiple effectors in the liver synthesize the ECM, HSCs are predominantly the primary source. HSCs necessitate activation through a complex network encompassing numerous fibrogenic and inflammatory pathways [67,68]. HSCs are activated by soluble stimuli including paracrine signals emanating from adjacent cell, such as macrophages, and oxidant stress signals like reactive oxygen intermediates [69]. This process leads to the transformation of quiescent HSCs (qHSCs), which are rich in vitamin A (VA), into cells that are devoid of VA droplets. The activation of HSCs is divided into 2 phases: initiation and perpetuation. aHSCs have a more vital ability to increase, contract, and release proinflammatory, profibrogenic, and promitogenic cytokines and interact with other liver cells involved in liver inflammation [70].

At the molecular level, the processes promoting fibrosis are widespread and intricate [67]. Since inflammation often occurs before fibrosis, inflammatory cytokines significantly impact fibrosis [70]. Macrophages play a key role in regulating dynamic fibrogenesis in the liver, producing and activating the archetypal profibrotic cytokine transforming growth factor β (TGF-β), which enhances the production of ECM and tissue inhibitor of metalloproteinase-1 by myofibroblasts. Additionally, hepatic macrophages can produce platelet-derived growth factor (PDGF), strongly stimulating myofibroblast proliferation. They also generate interleukin-1b (IL-1b), tumor necrosis factor-α (TNF-α), and several chemokines, which induce further aggregation of inflammatory cells, allowing proinflammatory and profibrotic stimuli to persist. However, macrophages also play a key role in the regression of fibrosis as they can support fibrosis regression by secreting MMPs to degrade fibrous scarring. The mechanism of this functional role, however, remains to be proven [71,72].

LSECs can stimulate the migration and recruitment of HSCs to vessels by synthesizing PDGF and TGF-β [73]. In conclusion, PDGF and TGF-β, synthesized by various cells, are the most essential growth factors for HSC activation and collagen production [70]. HSCs respond to multiple cytokines and growth factors, while aHSCs can also secrete inflammatory cytokines to stimulate their own activation [69]. The autocrine signaling of PDGF was one of the first cytokine circuits recognized in the activation of HSCs and remains one of the most potent signals [69,70]. TGF-β, a major profibrogenic cytokine, is activated by deposits in the ECM and expressed and released by various cell types [74]. Furthermore, HSCs might play a crucial role in the immune response within the damaged liver, directly secreting inhibitory cytokines or indirectly modulating immune cells to suppress hepatic inflammation and mediating tolerance [71,75]. aHSCs up-regulate intercellular adhesion molecule-1 (ICAM-1) and vascular cell adhesion molecule-1 (VCAM-1), leading to the recruitment and activation of lymphocytes [71]. On the other hand, apoptotic hepatocytes and dysfunctional cholangiocytes contribute to the progression of liver fibrosis through the production of oxidant stress signals such as reactive oxygen species (ROS) [69], which induce hepatocyte injury and death while inhibiting parenchymal cell proliferation [76].

In liver fibrosis, tissue hypoxia can stimulate the production of VEGF, a vital factor in promoting angiogenesis, through a typical pathway involving the transcription factor hypoxia-inducible factor 1α, triggering angiogenesis. Hypoxia-dependent angiogenesis often precedes or parallels fibrosis and has been suggested to possibly promote the formation of fibrotic septa, driving the development of liver fibrosis and ultimately leading to portal hypertension [73,76].

### Mechanism of collagen synthesis, oxidation, and cross-linking

In a healthy liver, low concentrations of ECM in the space of Disse facilitate efficient transportation of substances from the vascular lumen to liver cells, thereby contributing to maintaining function and integrity of liver cells [77]. Meanwhile, excess ECM components accumulate in the liver to form scar tissue [78]. Regardless of etiology, the molecular makeup of scar tissue in liver fibrosis remains consistent and comprises fibrous proteins (including fibrillary collagens, mainly type I and III collagen, and elastin) and glycoproteins (including fibronectin, proteoglycans, and laminin) [1]. Collagen accounts for a significant proportion of these, with a 4- to 7-fold increase [79].

**Fig. 3.**
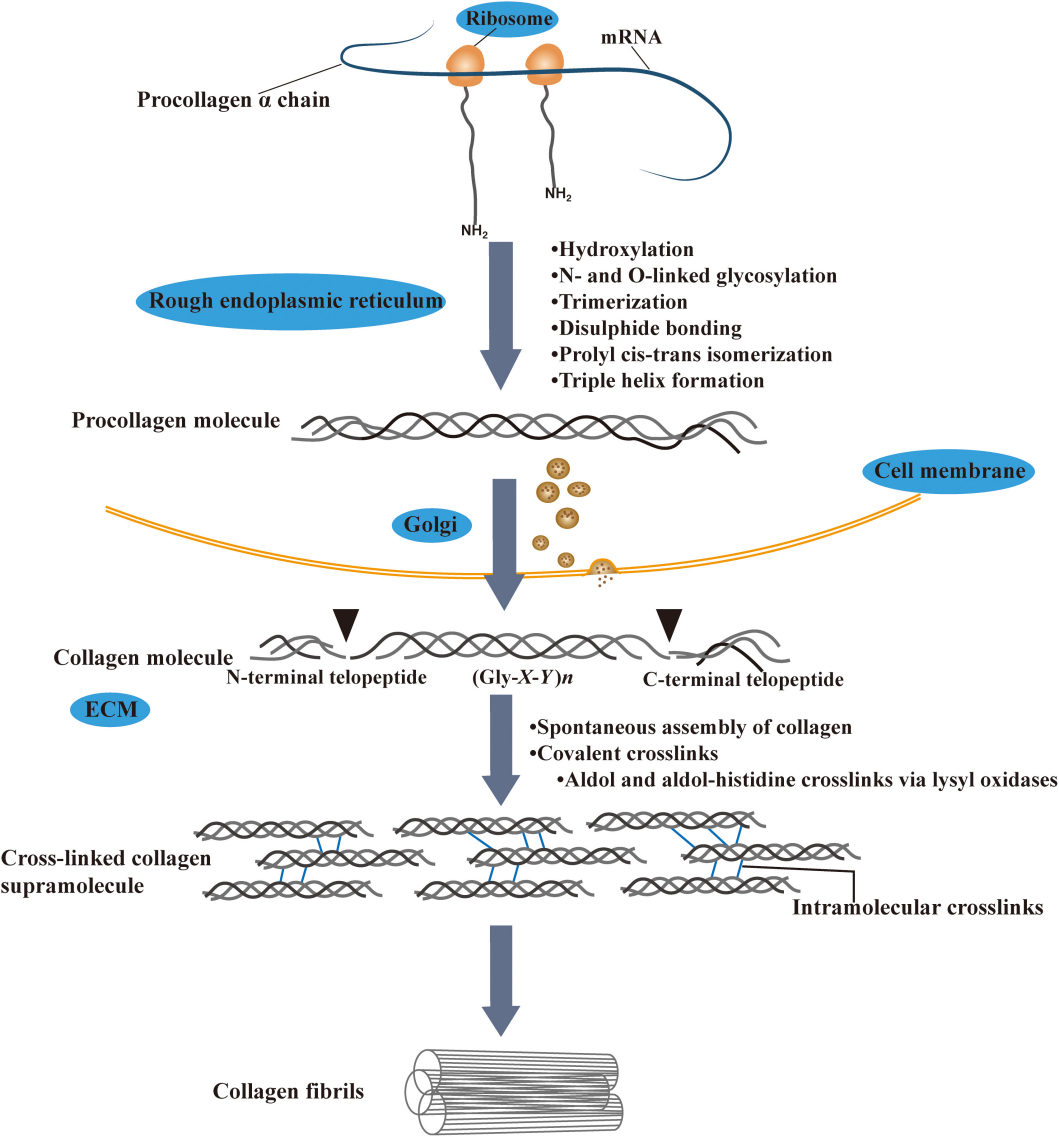
Schematic representation of type I collagen synthesis and covalent cross-linking formation.

Each type of collagen is composed of 3 homologous or heterologous α chains. A shared structural feature is a lengthy central triple-helical region in each α chain. This region is formed of consecutive (Gly-*X*-*Y*)*n* repeats, where *X* and *Y* represent any amino acid, and the value of *n* ranges from 337 to 343, depending on the type of collagen [80]. In collagen types I, II, and III, this region is bordered by short nonhelical regions called telopeptides, typically around 20 residues in length, found at both the N and C termini. Type I collagen, the primary type in hepatic fibrosis [81], is a heterotrimeric molecule consisting of 2 identical α1 chains and a distinct α2 chain, denoted as [α1(I)]_2_α2(I). The synthesis of type I collagen proceeds as follows. After transcription and translation, proα chains are transported into the rough endoplasmic reticulum, where they undergo several posttranslational modifications [82]. These modifications include hydroxylation of proline and lysine residues, N- and O-linked glycosylation, trimerization, disulfide bonding, prolyl cis-trans isomerization, and triple helix folding, which lead to the assembly of procollagen. Procollagen molecules are transported through the Golgi network and packaged into secretory vesicles before being exported to the ECM. The removal of the N- and C-terminal propeptide domains of procollagens triggers spontaneous collagen fibril assembly. Finally, the last stage of collagen biosynthesis entails the incorporation of covalent crosslinks, which stabilize the assembly within the ECM [83] (Fig. 3).

In the fibrotic liver, the expression of lysyl oxidase (LOX) family members, particularly LOX, LOXL1, and LOXL2, increases with the progression of fibrosis. Their increased activity stabilizes collagen through covalent cross-linking, making fibrotic scarring irreversible and enhancing the stiffness of the ECM. This increased stiffness further triggers the activation of fibrogenic effector cells, mainly aHSCs [84]. The cross-linking initiator enzymes in the LOX family oxidize lysine and hydroxylysine residues in the N- and C-terminal telopeptide regions, converting them into corresponding peptidyl aldehydes known as allysine and hydroxyallysine [85]. Once formed, aldehydes spontaneously form Schiff bases with ε-amine groups from unreacted lysines and hydroxylysines in other molecules to form major intermolecular crosslinks. Two allysyl residues can also form intramolecular crosslinks through a hydroxyaldehyde condensation reaction. Remarkably, the synthesis of crosslinks seems to be regulated mainly by the molecular arrangement in the collagen fibril without the need for additional enzymes [86,87]. Furthermore, the involvement of additional amino acid residues, such as peptidyl histidine, allows for the further maturation of these crosslinks into multivalent structures, ultimately leading to stable type I collagen [83].

## Potential Targets of Liver Fibrosis

Specific molecular and cellular processes are utilized as biochemical targets by novel molecular contrast agents to diagnose and stage liver fibrosis or to assess treatment efficacy [6]. The imaging of liver fibrosis involves exploring various targets, such as aHSCs, macrophages, inflammation markers, and ECM proteins like overexpressed type I collagen and synthesized oxidized collagen [38].

### Targeting of cells

#### Targeting of aHSCs

When the liver is injured, aHSCs play a central role in hepatic fibrosis, contributing to the inflammatory response, fibrogenesis, and angiogenesis. aHSCs transform into myofibroblasts, synthesizing and secreting ECM, mainly type I collagen. This leads to the disruption of liver tissue structure and function, serving as the underlying cause of liver fibrosis. Consequently, aHSCs represent a defining characteristic of liver fibrosis and are recognized as a significant imaging target [41]. Structures on aHSCs that can be developed as targets include integrin α_v_β_3_, vimentin and desmin, retinoic acid (RA)/VA receptor, PDGF receptor-β (PDGFR-β), and mannose 6-phosphate/insulin-like growth factor II receptor (M6P/IGF-IIR), among others. Ligands corresponding to these targets have also been developed for early diagnosis, prognostic prediction, and guiding and evaluating targeted therapy of aHSCs in liver fibrosis [88].

Integrin proteins, as widely expressed transmembrane glycoprotein receptors, serve as signaling proteins and adhesion receptors. They facilitate bidirectional signal transmission across the plasma membrane. When a ligand binds to an integrin receptor, it conveys extracellular information intracellularly, leading cells to respond to changes in the external environment. This response can lead to changes in gene expression, cytoskeletal structure, cell survival, cell polarity, and proliferation [89,90]. Of the numerous integrins discovered, integrin α_v_β_3_ has been the subject of extensive research. During liver fibrogenesis, aHSCs up-regulate integrin α_v_β_3_, while nonparenchymal cells like qHSCs, hepatocytes, and others exhibit lower levels of integrin α_v_β_3_ expression. Therefore, integrin α_v_β_3_ could be a selective target for molecular imaging of aHSCs in liver fibrosis [91]. When aHSCs bind to integrin α_v_β_3_ ligands that accumulate in the space of Disse, they may transmit proliferative and survival signals to aHSCs [92].

In addition, targeted imaging of integrin α_v_β_3_ allows for visualization of therapeutic strategies aimed at reducing collagen production. This can be achieved by disrupting the interaction between integrin α_v_β_3_ and ECM [93]. Another option is to use small inhibitory RNA (siRNA) to suppress the expression of integrin α_v_ or to use α_v_β_3_ disintegrin echistatin or neutralizing antibodies to dissociate α_v_β_3_, which is helpful for inhibiting the proliferation of HSCs [94]. The Arg-Gly-Asp (RGD) sequence is a cellular attachment site for a wide array of adherent ECM, blood, and cell surface proteins and is recognized by integrin α_v_β_3_ in its adhesion protein ligands. Based on this, several RGD derivatives have been developed to target integrin α_v_β_3_ of aHSCs [95]. Li et al. [91] designed a SPECT radiotracer of ^99m^Tc-labeled cyclic RGD (cRGD). The average ratio of radioactivity between the liver and heart was markedly elevated 45 min after intravenous administration compared to control rats, with the highest levels observed in the advanced stage of liver fibrosis. Hence, the noninvasive differentiation of different stages of liver fibrosis using ^99m^Tc-labeled cRGD represents a potentially valuable approach to monitor HSC activity by imaging the expression of integrin α_v_β_3_.

Nevertheless, the clinical application of ^99m^Tc-cRGD in liver fibrosis imaging is hindered by its low sensitivity, primarily attributable to the significant accumulation of ^99m^Tc-cRGD in the kidneys and the interference caused by renal shadows in the uptake images of ^99m^Tc-cRGD [91]. To enhance the effectiveness of targeted imaging, the use of dimeric or multimeric cRGD peptides conjugated to integrin α_v_β_3_ can optimize their binding affinity. Two factors contribute to the higher integrin α_v_β_3_ binding affinity of dimeric or multimeric RGD peptides compared to monomeric RGD radiotracers (e.g., ^99m^Tc-cRGD). First, the binding of one RGD motif to integrin α_v_β_3_ significantly increases the local concentration of a second RGD motif in the proximity to integrin α_v_β_3_ sites. Second, dimeric or multimeric RGD peptides have the ability to bind to 2 or multiple adjacent integrin α_v_β_3_ sites simultaneously, resulting in a bivalent or multivalent effect [96,97]. The PET tracer, [^18^F]-Alfatide, has been introduced for imaging integrin α_v_β_3_. Compared to the control group, both the mild and severe mouse models exhibited a significant increase in the radioactivity ratio of the liver to blood, which serves as a PET index for quantifying fibrosis progression 30 min after intravenous administration. For the first time, data obtained from human samples have demonstrated that the expression of integrin α_v_β_3_ protein increases as fibrosis progresses in human tissues. These findings also indicated that the probe [^18^F]-Alfatide is highly sensitive in detecting the progression of fibrosis. Therefore, [^18^F]-Alfatide/PET imaging could potentially offer a noninvasive method to quantify hepatic α_v_β_3_ expression [98].

The expression levels of desmin and vimentin are elevated in aHSCs [99]. These proteins, members of the type III intermediate filament protein family, possess a rod II domain on the cell surface that plays a vital role in stabilizing cytoplasmic structures. It has been reported that desmin and vimentin exhibit lectin-like properties on the cell surface, binding to N-acetylglucosamine (GlcNAc) [100,101]. Zhang et al. [102] conducted a study to develop a small molecular radiotracer, ^99m^Tc-GlcNAc-PEI. This study employed GlcNAc as the targeting moiety for aHSCs, while PEI-1800 served as the functionalized group for ^99m^Tc labeling (Fig. S1A). The resulting radiotracer demonstrated favorable properties, including hydrophilicity, high radiochemical purity (>98%), and excellent stability. ^99m^Tc-GlcNAc-PEI was utilized for noninvasive SPECT/CT imaging to target desmin and vimentin in aHSCs (Fig. S1B). This radiotracer directly reflects the extent of liver fibrosis and can be employed to detect liver fibrosis in mice as early as 4 weeks after CCl_4_ treatment (Fig. S1C and D). Furthermore, clodronate liposomes were administered to treat liver fibrosis, leveraging their capacity to eliminate macrophages and reduce liver fibrosis. Liver uptake of ^99m^Tc-GlcNAc-PEI in fibrotic mice subjected to clodronate liposome treatment was notably lower compared to the untreated group (Fig. S1E and F). Therefore, owing to its specific targeting of desmin and vimentin in aHSCs, ^99m^Tc-GlcNAc-PEI demonstrates robust binding affinity and reduced kidney uptake. This characteristic renders it a potential tool for directly detecting liver fibrosis and assessing the effectiveness of antifibrotic drugs. The integration of SPECT/CT imaging offers the advantage of providing both anatomical structure via CT and functional information through SPECT images, holding promise as a potential strategy for noninvasive monitoring of the progression and prognosis of hepatic fibrosis in clinical practice.

Approximately 80% of the body’s total VA is stored in HSCs in the form of retinylesters [103]. RA, the active metabolite of VA, regulates gene expression critical to physiological processes such as cellular energy metabolism. The activation of retinoic acid receptors (RARs) and retinoid X receptors (RXRs), subfamilies of the nuclear receptor superfamily, primarily mediates these processes [104]. Especially during the developmental stage of liver fibrosis, aHSCs may overexpress RA receptors [103]. Previously, RA has been investigated for liver targeting and therapeutic delivery design. Tran et al. [105] developed a manganese (Mn)-based T1 contrast agent, Mn-porphyrin (MnTPPS_4_)/retinoic acid-chitosan ionic-complex (MRC) NPs. Among these, chitosan, a positively charged biopolymer [106], and MnTPPS_4_, a negatively charged T1 contrast agent, formed an ionic complex and then introduced RA, targeting liver fibrosis by binding to RARs and RXRs in the liver for imaging. MnTPPS_4_ was released from MRC NPs in a controlled manner for up to 24 h, thus extending the MRI time window. The research demonstrated that MRC NPs, exhibiting favorable stability and negligible cytotoxicity, preferentially accumulate in the liver. The MRC NPs exhibit significant potential as a T1 contrast agent, facilitating early diagnosis of liver fibrosis. Additionally, they function as a valuable physiological tool for actively targeting the liver [105].

Moreover, advancements have been made in developing particles loaded with drugs and specifically targeting VA receptors. These particles are specifically designed to treat liver fibrosis. Studies have shown that valsartan has a significant antifibrotic effect [107,108]. However, valsartan lacks high drug permeability; thus, liposomes can be utilized as nanocarriers for valsartan, coupled with VA, to enhance its permeability and targeting ability toward aHSCs. In a mouse model of liver fibrosis, treatment with VA-coupled valsartan-loaded liposomes (VLCs) reduced the levels of fibrogenic mediators. This treatment led to liver function tests approaching normal levels. As a result, VLC holds excellent promise as an effective antifibrotic treatment [109].

**Fig. 4.**
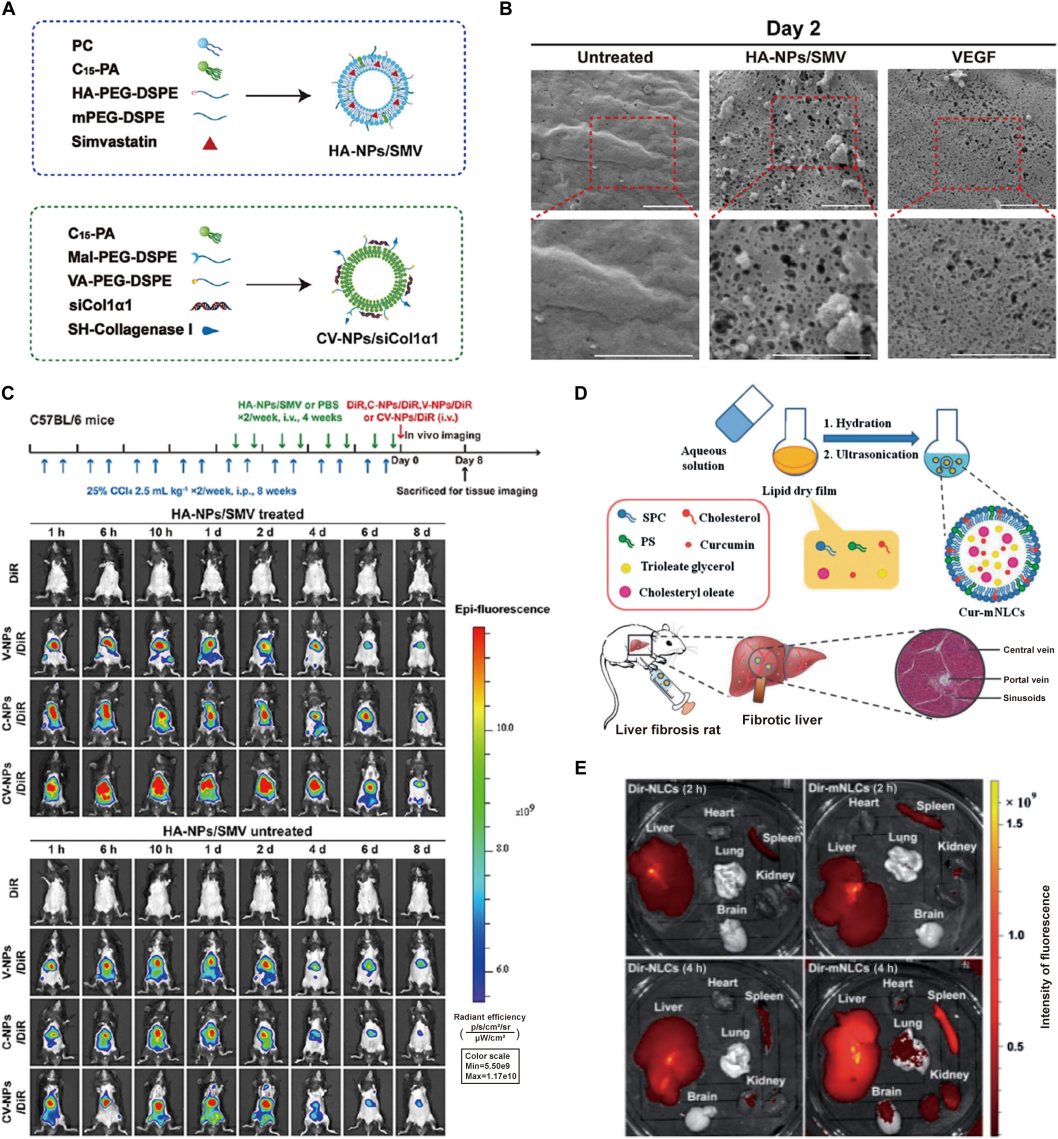
Targeting HA receptors on aHSCs and structures on macrophages for imaging liver fibrosis. (A) Schematic structure of HA-NPs/SMV and CV-NPs/siCol1α1. (B) SEM compares the number of fenestrations and sieve plates in primary LSECs and those treated with HA-NPs/SMV or VEGF. Scale bar, 1 μm. (C) Near-infrared fluorescence images of fibrotic mice treated or not with HA-NPs/SMV for 4 weeks followed by injection of indicated formulations. Reproduced with permission [110]. 2022, American Chemical Society. (D) Schematic representation of Cur-mNLCs synthesized and targeted to fibrotic liver. (E) In vitro fluorescence images of major organs of rats with liver fibrosis at 2 and 4 h after injection of DiR-loaded NLCs without PS (DiR-NLCs) or with PS (DiR-mNLCs). Reproduced under terms of the CC-BY license [126]. 2017 The Authors, Published by [Informa UK Limited].

In the process of liver fibrogenesis, the development of liver sinusoidal capillarization and the deposition of ECM create 2 concurrent pathological barriers that impede the precise targeting of VA at aHSCs and enhance drug delivery for liver fibrosis therapy. Zhang et al. [110] developed a strategy using a sequential nano-penetrator system to modulate these pathological barriers. HA-NPs/SMV, NPs targeting LSECs and promoting fenestrae repair, were developed based on corresponding hyaluronic acid (HA) modification and simvastatin (SMV) loading to address the first barrier. To overcome the second barrier, CV-NPs/siCol1α1, NPs combining VA and collagenase I, were designed to target HSCs and ablate collagen. Delivery of siCol1α1 through CV-NPs/siCol1α1 was aimed at inhibiting collagen production and deactivating HSCs (Fig. 4A). As shown in Fig. 4B, HA-NPs/SMV-treated primary LSECs exhibited more fenestrae and sieve plates than those treated with VEGF and untreated controls, indicating that HA-NPs/SMV enhanced NP penetration into the Disse space for improved therapeutic outcomes. In vivo studies using fibrotic mouse models and analyses of liver tissue sections showed that when confronted with the capillarized LSECs barrier, HA-NPs/SMV exhibited rapid release of SMV and functioned as a fenestrae repairer, which facilitated the increased entry of CV-NPs/siCol1α1 into the Disse space for degrading deposited collagen, and ultimately enabled VA-targeted delivery to aHSCs, resulting in enhanced siCol1α1 accumulation and reduced liver fibrosis (Fig. 4C). This sequential nano-penetrator system is capable of repairing LSEC fenestrae and degrading deposited collagen to increase drug accumulation, thereby facilitating deep delivery and precise targeting in the treatment of liver fibrosis. This research contributes to developing therapeutic strategies for liver fibrosis and effectively addresses the low treatment efficacy resulting from pathological barriers that prevent drug delivery and absorption [110].

In addition, numerous therapeutic targets of aHSCs are available in various preclinical studies. For example, Li et al. [111] engineered sterically stabilized liposomes (pPB-SSL) that incorporate a cyclic peptide pPB, enabling specific recognition of PDGFR-β present on the surface of aHSCs. Recombinant human TRAIL (rhTRAIL) protein, an essential protein for inducing apoptosis in aHSCs and treating liver fibrosis, was selected as the cargo within pPB-SSL and applied to treat liver fibrotic mice. The research findings demonstrated that the pPB-SSL delivery system effectively extended the blood circulation time of rhTRAIL and specifically targeted aHSCs—this approach successfully attenuated fibrosis both in vitro and in vivo. Besides, the up-regulation of M6P/IGFII-R on aHSCs during liver fibrosis has also been exploited as a targeting site. Hesperidin exhibits strong antifibrotic effects, but its oral bioavailability and targeting efficiency are limited. The carrier system of M6P–bovine serum albumin-conjugated hesperidin-loaded liposomes facilitates selective binding to M6P/IGFII-R. The particles can enhance efficacy by delivering the therapy in a sustained and cell-specific manner at an optimal rate, thereby targeting aHSCs and effectively attenuating liver fibrosis [112]. The targets mentioned above hold promise for imaging liver fibrosis and monitoring its therapeutic effect in future studies.

#### Targeting of macrophages

Hepatic macrophages are composed of resident macrophages (Kupffer cells) and monocyte-derived macrophages, which can be categorized as M1 or M2 macrophages. The M1/M2 phenotype of these macrophages determines their pro-inflammatory or anti-inflammatory effects, thereby influencing the progression of fibrosis in the later stages [113]. For example, Wei and colleagues [114–117] developed a multifunctional tetrahedral DNA nanoplatform (TDN-siTNF-α/-G4-MnP4), which can silence the expression of TNF-α in macrophages using TNF-α siRNA and polarize macrophages into the anti-inflammatory M2 phenotype. Importantly, this platform can eliminate intracellular ROS and promote hepatocyte proliferation, providing a synergistic and effective therapeutic strategy for acute liver failure.

Hepatic macrophages play a central role in the process of liver fibrosis, being responsible for sustaining inflammation and hepatocyte injury, as well as activating HSCs to promote fibrosis. When liver injury ceases, hepatic macrophages transition into reparative phagocytes, aiding in tissue repair and reducing fibrosis. In short, hepatic macrophages are key players in the pathogenesis of liver fibrosis [118]. Hepatic macrophages constitute the largest population of phagocytes in the body, which also are capable of directly interacting with NPs and other probes in the bloodstream. They possess stronger phagocytic abilities and multiple uptake pathways. Moreover, the liver receives a significant blood supply, and probes move slowly within the hepatic capillaries. Therefore, compared to inflammatory macrophages from other sites, liver macrophages exhibit a higher priority in probe uptake [119]. In addition, the influence of other inflammatory macrophages on liver fibrosis detection could be reduced by improving the probes [120]. Consequently, targeting of hepatic macrophages is a reliable method for detecting liver fibrosis. Similar targeting strategies have been successfully employed in the research of pulmonary fibrosis and atherosclerosis. For example, in atherosclerosis research, superparamagnetic iron oxides (SPIOs) coated with dextran or human ferritin protein cages can selectively target macrophages within vulnerable plaques, potentially allowing for noninvasive quantification of atherosclerotic plaque [121]. In pulmonary fibrosis research, a macrophage-targeted probe called ^64^Cu-BMV101 has been used for dual PET and optical imaging of pulmonary fibrosis in mice, while ^68^Ga-BMV101 has been used for PET imaging in patients with pulmonary fibrosis to monitor macrophage activity in both mouse models and human patients [122].

Translocator protein (TSPO), also known as the peripheral-type benzodiazepine receptor (PBR), is a nucleus-encoded transmembrane protein that selectively targets the mitochondria. Scientific research has established its significant involvement in regulating mitochondrial function [123]. During liver fibrogenesis, the number of TSPO-expressing macrophages and HSCs increases along with the fibrosis progression. [^18^F]FEPPA, a specific ligand labeled with ^18^F using PET techniques, exhibits a high affinity for TSPO. In the bile duct-ligated (BDL) rat model of liver fibrosis, the accumulation of [^18^F]FEPPA increases with the progression of liver injury and the expression levels of TSPO. Therefore, [^18^F]FEPPA can serve as sensitive probes for longitudinally monitoring liver fibrosis [124].

In addition, scavenger receptors expressed on macrophages also serve as targets [41]. Phosphatidylserine (PS) is an anionic phospholipid found in cell membranes, predominantly situated in the intracellular membrane of the phospholipid bilayer. Upon the occurrence of apoptosis, a substantial number of PS is translocated to the outer cell membrane. This exposed PS serves as a specific recognition signal for macrophages to phagocytize apoptotic cells [125]. Comprehensive research has showcased the potent antifibrotic effects of curcumin (Cur). Thus, PS-modified nanostructured lipid carriers (mNLCs) loaded with Cur (Cur-mNLCs) have been engineered to mimic apoptotic cells, thereby contributing to the treatment of liver fibrosis (Fig. 4D). Cur-mNLCs NPs were found to prolong the retention time of Cur in vivo and improve its bioavailability in rats with liver fibrosis. Cur-mNLCs NPs were shown to specifically target the liver, which is abundant in macrophages, as substantiated by both in vitro imaging and tissue distribution studies (Fig. 4E) [126]. Moreover, these NPs demonstrate the capability to mitigate liver fibrosis by enhancing the expression of hepatocyte growth factors and MMP-2. Cur-mNLCs improve the bioavailability of Cur in the liver and address several limitations associated with Cur that hampered its widespread clinical use. Based on these findings, it is anticipated that Cur-mNLCs NPs will emerge as a promising drug delivery system for treating liver fibrosis [126].

### Targeting of matrix deposition and cross-linking

#### Targeting of collagen

In response to tissue damage, HSCs are activated and differentiate into myofibroblasts, which produce structural proteins that form fibrous scarring in the area of injury. Among these, the excessive deposition of type I collagen is specific to fibrosis and is the most abundant collagen in vivo [38]. Notably, it holds a concentration of more than 10 μm in fibrotic tissues, and its extracellular location facilitates detection by probes, rendering type I collagen a highly compatible target with most imaging modalities [39].

Several probes, including EP-3533, CM-101, and ProCA32.collagen1, have been developed to enable molecular imaging of collagen deposited during liver fibrosis [39,127]. EP-3533 is a Gd-based probe comprising a cyclic peptide consisting of 10 amino acids with 3 Gd moieties linked together. This probe exhibits a high affinity for type I collagen (*K*_d_ =1.8 μm), and its Gd moieties demonstrate a significantly enhanced signal enhancement capability (relaxivity = 16.2 mM^−1^ s^−1^ at 4.7 T) compared to gadopentetate dimeglumine (Gd-DTPA, relaxivity = 3.8 mM^−1^ s^−1^ at 4.7 T) used as a control. In comparison to controls, EP-3533-enhanced MRI revealed higher signal intensity and prolonged signal retention during delayed imaging of the liver fibrosis rat model (Fig. S2A and B). This allowed differentiation between fibrotic and control animals. These results indicated that MRI utilizing a type I collagen-targeting probe EP-3533 can effectively differentiate liver fibrosis in animal models. However, it warrants emphasis that this preliminary feasibility study lacked the requisite power to accurately stage the extent of fibrosis [13].

To confirm the efficacy of EP-3533 in distinguishing different stages of liver fibrosis in mice, EP-3533-enhanced MRI was compared with other MRI techniques previously utilized for fibrosis detection, including relaxation times (T1, T2, T1ρ), diffusion, and magnetization transfer measurements. The most sensitive MRI measure for correlating the MRI signal with fibrosis was identified as the change in contrast to noise ratio (∆CNR) observed between the liver and adjacent skeletal muscle following the administration of EP-3533. After injection of EP-3533, ∆CNR was found to increase with the advancement of disease (Ishak score) in mice with liver fibrosis (Fig. S2C and D), and the area under the receiver operating characteristic (ROC) curve to differentiate between early and advanced fibrosis registered 0.942 ± 0.052. There were also strong correlations between ∆CNR and the levels of liver hydroxyproline (*r* = 0.89, Fig. S2E). In contrast, other MRI techniques showed lower sensitivity to detect fibrosis changes in this model [128].

EP-3533 has been shown to accurately assess the reduction of liver fibrosis after treatment. Prior research has demonstrated the efficacy of rapamycin in reducing fibrosis in a BDL rat model of liver fibrosis [129]. Farrar et al. [130] employed a respiratory-gated 3D inversion recovery MRI sequence to measure the change in longitudinal relaxation rate (∆R1) caused by the collagen-targeting probe EP-3533 throughout the entire liver in BDL rats. Previously, ∆CNR has been performed on only a few liver image sections, whereas ∆R1 potentially offers a more reliable and quantitative metric for assessing fibrosis and measuring disease heterogeneity in the liver than ∆CNR. EP-3533 was observed to markedly increase the ∆R1 with the disease progression compared to controls in vitro measures of increasing fibrosis (Fig. S2F to H) and to reduce the ∆R1 in animals treated with rapamycin compared to untreated controls (Fig. S2I). Thus, EP-3533 can potentially serve as a tool for staging liver fibrosis and evaluating the efficacy of drug therapy.

Similarly, EP-3533 can measure the antifibrotic impact of EDP-305, a novel agonist of the farnesoid X receptor [131]. Collagelin, a peptide that binds to collagen, was covalently attached through click chemistry to pegylated ultrasmall superparamagnetic iron oxide (USPIO) NPs, forming USPIO-PO-PEG-Collagelin NPs. The NPs demonstrate MRI detectability at low concentrations that simulate the early stages of fibrosis. In collagen hydrogels, the signal diminishes in MRI as the collagen concentration increases. Furthermore, USPIO-PEG-PO-Collagelin NPs prove to be biocompatible and do not result in accumulation in cells like fibroblasts and macrophages, making them suitable for various medical applications. The effectiveness of USPIO-PEG-Collagelin NPs as nanotracers for molecular imaging using MRI has been widely recognized [132].

#### Targeting of oxidized collagen as a marker of fibrogenic activity

During liver fibrogenesis, LOX and LOX-like enzymes experience up-regulation and oxidize collagen lysine ε-amino groups to aldehydes (allysine). Due to the abundant substrate concentration and effective catalytic activity of LOXs, in combination with the slow rate of subsequent condensation reactions into crosslinks between proteins, there is an accumulation of extracellular allysine during fibrogenesis. Therefore, extracellular allysine of oxidized collagen could represent a particular target for fibrogenesis [39,127,133]. Allysine is known to react readily with nucleophiles, including hydrazines, hydrazides, and oxyamino groups, forming more stable but reversible condensation products [39].

**Fig. 5.**
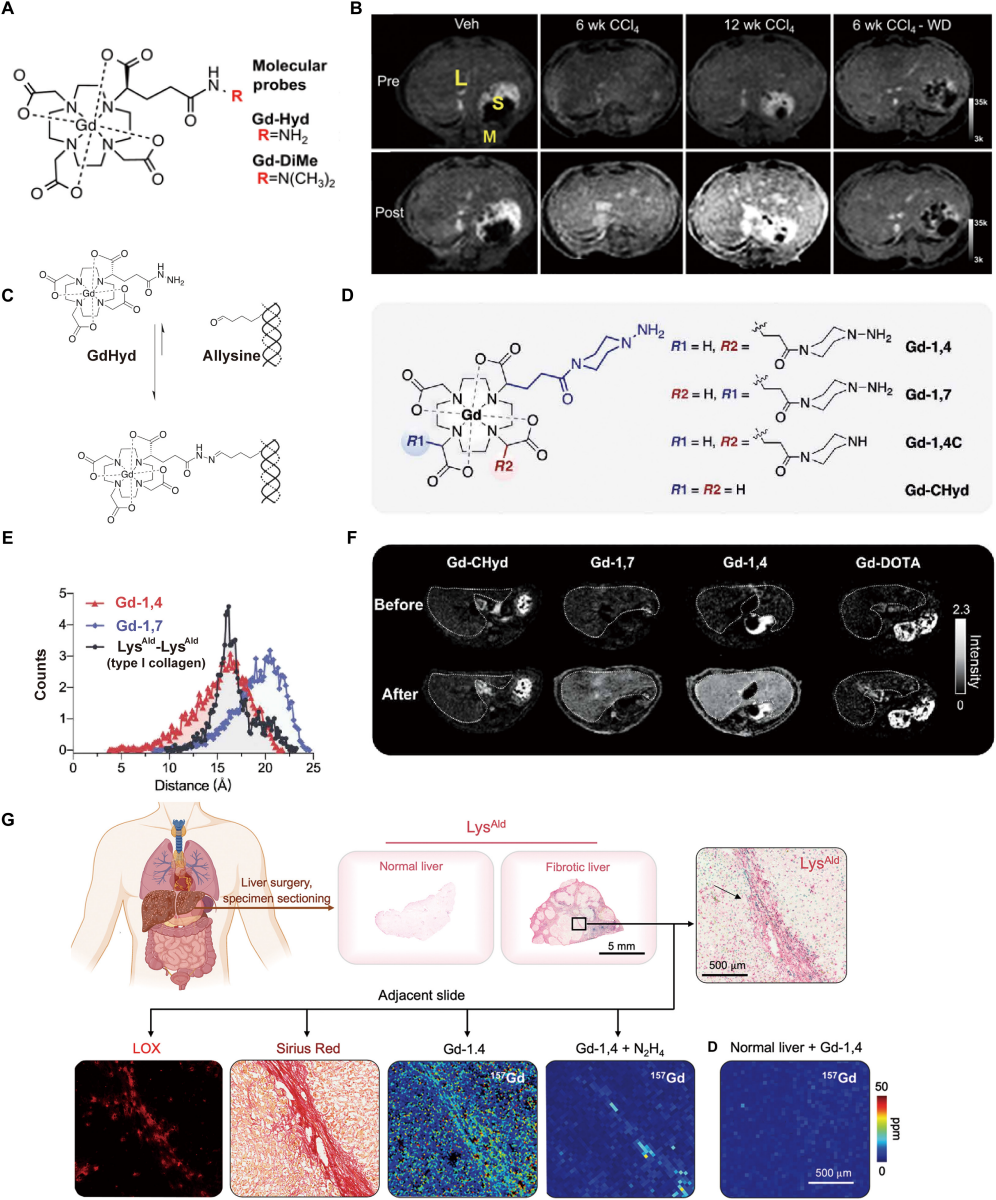
Assessment of fibrogenesis using probes targeting oxidized collagen. (A) Schematic structure of Gd-Hyd and Gd-DiMe (control). (B) MRI-enhanced imaging of liver fibrosis progression and regression in hepatic fibrosis mice before and after Gd-Hyd injection compared to controls. Reproduced with permission [135]. 2017, American Society for Clinical Investigation. (C) Schematic representation of the binding process of the hydrazide moiety on Gd-Hyd to allysine on collagen. Reproduced under terms of the CC-BY license [134]. 2021 The Authors. (D) Schematic chemical structures of Gd-1,4, Gd-1,7, Gd-1,4C, and Gd-CHyd. (E) Compared with Gd-1,7, the O-O distance between 2 Lys^Ald^ residues in type I collagen is more consistent with the N-N distance in piperazino-hydrazine groups in Gd-1,4. (F) Liver MRI of CCl_4_ mouse before and after injection of different probes. (G) Translation potential of Gd-1,4 in detecting human liver fibrogenesis. Reproduced with permission [137]. 2022, The American Association for the Advancement of Science.

The probe Gd-Hyd was designed by conjugating the gadoterate meglumine (Gd-DOTA) chelate with the hydrazide group, which can undergo a reversible condensation reaction with aldehydes to produce hydrazone. This design enables Gd-Hyd to target the elevated micromolar levels of allysine observed during active fibrosis specifically (Fig. 5A and C) [134,135]. To minimize nonspecific protein binding and facilitate rapid renal excretion, Gd-Hyd was deliberately designed to possess small size, anionic charge, and high hydrophilicity. In comparison with the negative control Gd-DiMe (Fig. 5A), Gd-Hyd shows specificity in mice treated with CCl_4_. It has shown that Gd-Hyd-enhanced MRI is capable of detecting liver fibrosis, monitoring the progression of the disease, and assessing the regression of fibrosis following toxin withdrawal (Fig. 5B) [135]. In addition, the fibrogenesis probe Gd-Hyd was employed to gain further insights into the activity of fibrotic disease and the presence of steatosis in a mouse model of NASH. Gd-Hyd signal enhancement enabled the differentiation between active fibrogenesis and stable scar formation. It exhibited a robust association with LOX expression and functioned as a dependable marker for assessing the degree of active fibrotic disease progression. This has demonstrated the potential of Gd-Hyd imaging in monitoring treatment response of liver fibrosis [134].

During liver fibrogenesis, LOX catalyzes the cross-linking of collagen through the selective oxidation of adjacent lysine amino pairs to allysine aldehyde (Lys^Ald^) pairs [136]. Compared to a monobinder previously used, the dual-binding approach showed improved performance, with a 10-fold higher ∆CNR, in accurately measuring liver fibrogenesis in vivo. Ning et al. [137] conducted a study in which they incorporated 2 piperazino-hydrazine moieties onto the α-carbons of the 2 Gd-DOTA carboxylate arms. This chemical modification yielded 2 compounds: cis-1,4-Gd-(CHyd)_2_ (Gd-1,4) and trans-1,7-Gd-(CHyd)_2_ (Gd-1,7), which have different distances between the 2 hydrazine moieties. Two additional control compounds, Gd-1,4C and Gd-CHyd, were prepared (Fig. 5D and E). In vitro studies indicated a potential preference for Gd-1,4 for targeting Lys^Ald^ pairs. Gd-1,4 did not bind nonspecifically and did not accumulate in normal tissues, which led to its rapid elimination from the kidneys. They further validated the exceptional sensitivity and specificity of Gd-1,4 in a mouse model of liver fibrosis as well as in human fibrotic liver tissue. In CCl_4_-injured mice, there was a difference in ∆CNR among the probes, with a ranking of Gd-1,4 > Gd-1,7 ≈ Gd-CHyd > Gd-DOTA, which may be attributed to their varying affinities with Lys^Ald^ (Fig. 5F). There was a strong correlation between quantitative molecular MRI and fibrogenic markers. In human liver specimens, Lys^Ald^ staining showed elevated levels in fibrotic regions of the liver, while negligible or minimal staining was observed in nonfibrotic liver samples (Fig. 5G). Gd-1,4 shows excellent promise for being translated into clinical applications. The production of Gd-1,4 involves a straightforward synthesis comprising just 5 steps and achieves a high overall yield of over 50%. When fibrosis is active, the levels of Lys^Ald^ increase. However, if fibrogenesis ceases, the breakdown of aldehydes would be expected, indicating that this approach can be used for early disease detection and for measuring the response to treatment success [137].

## Molecular Imaging Probes

Noninvasive molecular imaging probes, also referred to as contrast agents, tracers, and molecular beacons, are labeled compounds that possess a high specificity for binding to molecules participating in biological pathways or relevant to diseases. These probes can be tracked both in vivo and in vitro, allowing for the visualization of the functions of their target molecules and the generation of image signals for identification [138,139]. The use of high-affinity probes to identify and validate targets is a prerequisite for studying specific molecular targets. Probes can include small molecules like receptor ligands or enzyme substrates, as well as larger high-affinity ligands like recombinant proteins, monoclonal antibodies, and NPs [140]. A molecular imaging probe typically comprises 3 components: a signal agent, a targeting moiety, and a connecting linker. The signal agent is responsible for generating signals for various imaging modalities including magnetic molecules (MRI), radionuclides (PET and SPECT), fluorescent molecules (optical imaging), and microbubbles (MBs) (US), among others [37]. Ideal molecular probes for liver fibrosis should be targeted toward liver fibrosis to achieve a lower background signal. For staging, the precision of the measurement must be high enough to accurately differentiate signal changes at different fibrosis stages [38]. This section will provide an overview of the molecular imaging probes currently used for diagnosing and monitoring disease progression and detecting treatment response to liver fibrosis. We will discuss the constituent materials, targeting ligands, cell/molecular targets, and imaging methods of these probes (Table).

**Table.** Molecular imaging probes for liver fibrosis

Materials	Targeting ligand	Molecular/ cell target	Imaging method	Additional Information	References
EP-3533	Peptide	Type I collagen	MRI(T1)	High sensitivity High specificity Versatility Rapid elimination Minimal nonspecific binding Low stability Risk of gadolinium retention [13,128,130,131,241,242]	
CM-101	Peptide	Type I collagen	MRI(T1)	High chemical stability Fast blood clearance Rapid renal elimination Low background signal	[153]
ProCA32.collagen1	Peptide	Type I collagen	MRI(T1/T2)	High sensitivity High specificity Stability No gadolinium retention High relaxivity Greater metal selectivity	[154]
Gd-Hyd	Hydrazide	Allysine	MRI(T1)	High sensitivity Stability Minimal nonspecific binding Rapid and complete renal elimination Simplicity Suitable for large-scale synthesis	[134,135]
EP-2104R	Peptide	Fibrin	MRI(T1)	High sensitivity Rapid blood clearance Low nonspecific binding	[243]
Gd-1,4	Piperazino-hydrazine moieties	Lys^Ald^ pairs	MRI(T1)	High sensitivity High specificityv Faster on-rates Slower off-rates Higher relaxivity Versatility Rapid blood clearance Rapid renal elimination Low nonspecific binding	[137]
Gd-P	CLT1	Fibronectin	MRI(T1)	High specificity Rapid elimination	[244]
Gd-ESMA	-	Elastin	MRI(T1)	High specificity	[245]
Den-RGD	RGD	Integrin α_v_β_3_	MRI(T1)	High specificity Unaffected by hepatocyte injury Nonspecific uptake	[246]
MRC NPs	RA	RARs	MRI(T1)	High specificity Prolonged imaging time window Stability	[105]
IO-DyO NPs	PAA	Type I collagen	MRI(T2)	High sensitivity High specificity Biocompatibility Biosafety Highest accumulation in the liver Distribution hindrance by fibrosis tissues or inflammation	[164]
c(RGDyC)-USPIO	c(RGDyC)	Integrin α_v_β_3_	MRI(T2)	High specificity	[166,247]
cRGD-PLGA/IOFA	cRGD	Integrin α_v_β_3_	MRI(T2)	High sensitivity High specificity Biosafety	[240]
D-SPIONs	Dextran	ASGPR	MRI(T2)	Long circulation time High relaxivity Enhanced magnetic saturation Biocompatibility Stability	[248]
C-USPIONs	-	-	MRI(T2)	Aqueous dispersibility Stability Biocompatible Higher relaxivity	[163]
USPIO-PO-PEG- Collagelin NPs	Collagelin	-	MRI(T2)	High specificity Stability Low cytotoxicity	[132]
HTCDGd	Oxyamine	Allysine	MRI(T1)/FI	High sensitivity High specificity High relaxivity Biosafety Stability Rapid blood clearance Prolonged imaging time window	[249]
[^52^Mn]Mn-2CHyd	Piperazino- hydrazine moieties	Lys^Ald^	MRI(T1)/PET	High sensitivity High specificity High relaxivity Stability Rapid blood clearance Low nonspecific binding	[172]
P-SPIONs	Pullulan	ASGPR	MRI(T2)/NIR	High specificity Stability High relaxivity Biocompatible	[250]
SPIO@ SiO_2_–ICG–RGD	RGD	Integrin α_v_β_3_	MRI(T2)/NIR	High specificity Stability Biocompatible Rapid clearance Nonspecific uptake	[251]
cRGD-PLGA-Fe_3_O_4_ PFOB NPs	cRGD	Integrin α_v_β_3_	MRI/US/CT	High specificity Real-time imaging Biocompatible	[252]
^99m^Tc-GlcNAc-PEI	GlcNAc	Desmin and vimentin	SPECT/CT	High radiochemical purity High specificity Stability Nonspecific uptake	[102]
^99m^Tc-3PRGD2	RGD	Integrin α_v_β_3_	SPECT/CT	High specificity High sensitivity Minimal nonspecific binding	[191]
^99m^Tc-CBP1495	CBP1495	Type I collagen	SPECT/CT	High radiochemical purity Rapid blood clearance Low nonspecific binding Stability Short circulation time	[253]
^99m^Tc labeled cRGD	cRGD	Integrin α_v_β_3_	SPECT/CT	High radiochemical purity Nonspecific uptake Low sensitivity	[91]
^99m^Tc-MAMA-MGal	MAMA-MGal	ASGPR	SPECT/CT	High radiochemical purity High specificity Rapid hepatobiliary system and renal clearance	[254]
[^18^F]FEDAC	-	TSPO	PET/CT	High radiochemical purity High sensitivity Overlapping imaging features between fibrosis with hepatitis	[123,181]
Al[^18^F] F-NOTA-PEG_3_- Duramycin	Duramycin	PE	PET/CT	Rapid renal elimination	[179]
[^18^F]-Alfatide	RGD	Integrin α_v_β_3_	PET/CT	High specificity High sensitivity Nonspecific uptake	[98]
[^18^F]fluoro-proline	Proline	HSCs	PET/CT	Nonspecific uptake	[255]
[^18^F]MAGL-4-11	MAGL inhibitor	MAGL	PET/CT	High specificity High sensitivity	[256]
^18^F-FBHGal	FBHGal	ASGPR	PET/CT	High radiochemical purity Rapid clearance High specificity Stability	[257]
[^18^F]PBR06	-	TSPO	PET/CT	High specificity Ease of preparation	[258]
[^68^Ga]GSA	GSA	-	PET/CT	High sensitivity	[259]
[^68^Ga]Ga-NO2A-[Nle^13^]-Col	Collagelin analog	Type I collagen	PET/CT	High radiochemical purity Rapid clearance Stability Nonspecific uptake	[184]
[^68^Ga]Ga-BOT5035	BiPPB	PDGFR-β	PET/CT	Rapid clearance Stability Nonspecific uptake	[260]
[^68^Ga]Ga-DOTA-PEG_2_-LRELHLNNN	LRELHLNNN	Type I collagen	PET/MRI	Rapid clearance Nonspecific uptake Limited sensitivity	[183]
^18^F-FEPPA	-	TSPO	PET/MRI	High sensitivity High specificity	[124]
NML	Leucine/ Monoamine	LAP/MAO	NIR FI	Higher specificityBiocompatible	[261]
PEI-D-GlcNAc-ICG	GlcNAc	Desmin and vimentin	NIR FI	Low toxicity High specificity Long-lasting fluorescence signal	[239]
C-TPP-tagged collagelin	Collagelin	Type I collagen	NIR FI	Highest accumulation in the liver High specificity	[262]
DHMP2	Propylamine	MAO-A	NIR FI	High quantum yield Good cell permeability Stability High selectivity High specificity Biocompatibility	[198]
HA-QDot conjugates	HA	HA receptor	NIR-I FI	Long-lasting deliveryHigh specificity	[263]
SCH4	-	-	NIR-II FI	Rapid renal elimination High tumor/normal tissue ratio	[199]
LnNRs@ mSiO_2_-RBS	-	-	NIR-II FI	High specificity	[200]
IP	cRGD	Integrin α_v_β_3_	FI/PAI	High specificity High sensitivity Deeper penetration depth Real-time imaging Biocompatibility Good solubility	[227]
BiPhAA	Benzylamine	MAO-B	TP FI	High specificity High sensitivity Simplicity Fast imaging Real-time imaging Photostability Biocompatibility	[264]
cRGD-PLGA-PFOB NPs	cRGD	Integrin α_v_β_3_	US	Stability Low cytotoxicity Biocompatibility Prolonged imaging time Stronger penetrability	[216]
ICG	-	-	DCE-PAI	Deeper penetration depth Low photostability Low specificity Photo bleaching	[206]
GLTTs	GA	GA-R	SERS	Biocompatibility Stability Reproducibility	[221]
LTGNDs	GA	GA-R	SERS	High sensitivity Biosafety	[265]

## Available Molecular Imaging Modalities for Liver Fibrosis

Molecular imaging can utilize conventional diagnostic imaging methods like MRI, CT, PET, and others and incorporates molecular imaging probes to identify the expression of specific molecular markers at different stages of liver fibrosis [37]. An extensive range of targeted probes have been developed, conjugated with varying labeling moieties for imaging. As imaging technologies evolve, there will be a growing demand for more advanced contrast agents. NPs are especially well suited to addressing this demand due to their capacity to integrate numerous imaging agents into a single platform in a stable manner [33].

### MRI

MRI is one of the most commonly used diagnostic modalities in clinical practice due to its noninvasiveness, absence of ionizing radiation, and excellent penetration depth while providing higher spatial resolution than other clinical imaging methods [141]. MRI displays radio frequency (RF) signals that are emitted by tissue during the image acquisition process, of which the basis is the interaction between RF pulses and magnetic moments of ^1^H atoms [142]. In clinical diagnosis, a sample is exposed to a strong magnetic field, typically 1.5 or 3 T, causing the magnetic moments of protons to align and creating an equilibrium magnetization along the longitudinal axis. RF pulses, operating at a resonant frequency of 5 to 100 MHz, can then rotate the magnetic moments of protons from the longitudinal axis to a specific angle called the flip angle by transferring energy to the protons. Once the radiation is removed, the magnetic moments of the protons return to their equilibrium state. In MRI, this process is swiftly repeated with successive RF pulses [143]. In summary, ^1^H atoms transition from the excited state to the ground state when the RF pulses are removed, producing signals that can be used to create images [65].

As stated above, endogenous contrast is determined by the physical and chemical properties of the tissue itself, i.e., local differences in proton density, which give rise to different T1 and T2 values. While the tissue images are generally of good quality, there are situations where the endogenous image contrast is insufficient to diagnose the lesion of interest [144]. Since healthy and diseased tissues have different metabolisms and varying contrast agent uptake patterns, T1 and T2 can be selectively shortened to enhance the MRI contrast by introducing exogenous contrast agents to better differentiate between normal and diseased tissues. T1-weighted images effectively display anatomical structures and are preferred when clear structural images are required. T2-weighted images provide valuable pathological information as areas of abnormal fluid accumulation appears brighter than the surrounding normal tissue [145]. While most MRI contrast agents impact both T1 and T2, their effect on either T1 or T2 is typically more significant, resulting in their classification as either T1 or T2 contrast agents. Most existing T1 contrast agents are paramagnetic complexes, whereas most T2 contrast agents are primarily composed of SPIOs [146]. Next, we classify the available MRI contrast agents for MRI in liver fibrosis into 3 main categories: Gd-based, iron oxide-based, and Mn-based.

#### Gd-based contrast agents

Paramagnetic Gd-based contrast agents are introduced to further enhance anatomical features and improve diagnostic accuracy. Gd speeds up the T1 relaxation of water protons, leading to brighter MRI signals in T1-weighted MRI, which makes it one of the most frequently used MRI contrast agents in medical examinations [147]. All Gd complexes approved for clinical use consist of a single Gd^3+^ chelated with a low molecular weight acyclic or cyclic ligand such as DTPA, DOTA, and DO3A. Macrocyclic ligands based on cyclens, such as DOTA and DO3A, are favored over linear ligands like DTPA because of their enhanced stability and convenience for functionalization [148,149]. Due to their compact size, numerous agents of this kind disperse throughout the intravascular and interstitial spaces. They are swiftly eliminated through renal filtration, which may limit their continued application in liver fibrosis imaging [149].

On the other hand, if a patient has impaired renal function [glomerular filtration rate (GFR) <30 ml/min], the elimination half-life of Gd-based contrast agents will be extended in a manner that is inversely correlated with GFR [150]. Consequently, the reduced excretion rate in patients with renal failure allows time for Gd chelates to dissociate in vivo, resulting in the release of free Gd^3+^, which has been implicated in the development of nephrogenic systemic fibrosis in individuals with renal dysfunction [151]. Although Gd^3+^ is toxic to biological systems, it can be bound by appropriate ligands or chelates to form biologically available and nontoxic complexes [147], which are recognized today as MRI contrast agents with an outstanding safety profile and play a vital role in disease diagnosis and clinical medicine globally [152].

Gd-based contrast agents are also gradually being investigated for use in the MRI of liver fibrosis. Farrar et al. [153] reported a novel MRI probe, CM-101, which uses a more stable macrocycle Gd-DOTA chelate and utilizes a type I collagen-targeting mechanism for imaging hepatic fibrosis (Fig. S3A). After CM-101 injection, mice with liver fibrosis showed higher enhancement than control mice (Fig. S3B). Following CM-101 injection, the area under the curve (AUC) and ∆CNR of CCl_4_-treated mice were substantially elevated compared to baseline values, suggesting that CM-101 can robustly detect fibrosis (Fig. S3C and D). CM-101 showed rapid blood clearance and systemic elimination, with minimal accumulation of Gd in tissue or bone.

Furthermore, numerous NPs and macromolecules, such as dendrimers, proteins, polymers, vesicles, and micelles, have already been investigated as platforms for labeling and encapsulating Gd. Although promising, early work with Gd-labeled macromolecules has shown that these larger agents are excreted slowly and, in some cases, may hinder their translation to clinical application. Compared to small Gd complexes, the more prolonged circulation and retention time of NPs in patients underscores the importance of achieving a delicate balance between sufficient circulation time for effective targeting and rapid excretion. This balance is essential for developing safe and effective Gd-based NPs as MRI contrast agents for molecular imaging, aiming to minimize potential toxic side effects [149].

Salarian et al. [154] designed an MRI contrast agent for hepatic fibrosis imaging, ProCA32.collagen1, by incorporating a type I collagen-targeting peptide moiety at the C terminus of protein contrast agent ProCA32 (containing 2 Gd^3+^ binding sites), which has a strong affinity for type I collagen (Fig. S3E)—by leveraging the high R1 and R2 of ProCA32.collagen1, it was possible to quantify different periods of liver fibrosis and NASH across diverse mouse models (Fig. S3F to H). In addition, ProCA32.collagen1 was able to identify the heterogeneity of liver fibrosis and track the regression and treatment progress in fibrotic mice. Significantly, ProCA32.collagen1 exhibits enhanced resistance to transmetallation, along with selective binding to Gd^3+^, effectively reducing metal toxicity. The research findings also highlighted the serum stability of ProCA32.collagen1 and its lack of Gd^3+^ deposition in the brain. Thus, it is anticipated that ProCA32.collagen1 will address significant clinical challenges by enabling early diagnosis, noninvasive detection, and staging of liver fibrosis, and it has the potential for translational use in humans for monitoring treatment effectiveness.

#### Iron oxide-based contrast agents

In recent years, iron oxide-based contrast agents widely used for liver fibrosis are mainly iron oxide NPs because of their robust ability to reduce the T2 relaxation time in the surrounding area, leading to negative enhancement effect on T2 and T2^*^-weighted sequences [155]. Iron oxide NPs are categorized based on their size, including micrometer-sized paramagnetic iron oxide (MPIO) with dimensions in the micrometer range, SPIO particles with an average diameter exceeding 50 nm, and USPIO particles with smaller hydrodynamic diameters. SPIO and USPIO are presently employed for clinical imaging [156].

Iron oxide NPs are absorbed explicitly by macrophages in the liver and spleen, and this uptake by macrophages is independent of cell activation [157]. In inflammatory diseases such as fibrosis, macrophages absorb iron oxide NPs and then aggregate, causing a dark signal in a T2/T2^*^ sequence. Conversely, noninflammatory tissue does not show any contrast modification as it lacks macrophages, which creates a sharp opposing contrast to diseased tissues [158]. Importantly, iron oxide NPs are processed into a soluble and nonsuperparamagnetic form of iron via endogenous iron metabolism pathways, which is integrated into body’s normal iron reserves, such as ferritin, hemoglobin, and hemosiderin, within a few days, or excreted via the kidneys [159], making iron oxide NPs highly biocompatible. However, iron oxide NPs may also be degraded into ferric ions at low pH and in hydrolytic enzyme-catalyzed reactions within lysosomes. Hydroxyl radicals produced by free iron via the Fenton reaction can damage DNA, proteins, polysaccharides, and lipids in vivo [160]. In addition, there may also be difficulty in distinguishing a hypointense contrast effect of bleeding or calcification by SPIOs [161]. Iron oxide NPs generally consist of a core of iron oxide (mainly magnetite) and a coating of organic compounds [162]. Their effectiveness is influenced by several factors, including the size of the iron oxide crystals, the composition of the coating, the charge, and the hydrodynamic size of the coated particles [155].

To achieve optimal particle size and magnetic properties, Saraswathy et al. [163] opted to use citrate as a stabilizer for the USPIONs, ensuring high water dispersibility and long-term stability. After optimizing the coating thickness, the transverse to longitudinal relaxivity (R2/R1) ratio of NPs was as high as 37.92. A noticeable dose-dependent contrast enhancement was observed in T2-weighted MRI of a rodent liver fibrosis model. Thus, C-USPIONs with optimized parameters can produce effective MRI contrast for imaging liver diseases. On the other hand, their chemical coating is linked to molecules of the targeted ligands, making them highly sensitive for molecular imaging applications [148]. To date, several iron oxide NPs have been documented for the early diagnosis and staging of liver fibrosis. Balachandran et al. [164] developed small (4 nm) heterogeneous iron oxide/dysprosium oxide NPs (IO-DyO NPs) by mixing paramagnetic dysprosium with iron and then capping it with poly(acrylic acid) (PAA) to enhance their biocompatibility and physiological stability as a diagnostic contrast agent in ultrahigh-field MRI (7.0 and 9.4 T) for in vivo imaging of liver fibrosis [164] (Fig. S4A). Among them, the paramagnetic dysprosium and iron mixture could enhance the MRI signal, while PAA could target type I collagen by interacting with it. The slope R2 values of IO-DyO NPs in solution were 96.7 and 142.7 mM^−1^ s^−1^ at 7.0 and 9.4 T, respectively (Fig. S4B). In a liver fibrosis model of mice, IO-DyO NPs notably increased the MRI signal of the liver, resulting in a clear distinction between normal and fibrotic tissues (Fig. S4C), and the signal peaked within 1 h and remained stable for nearly 24 h (Fig. S4D). Moreover, IO-DyO NPs could further stage liver fibrosis. As liver fibrosis advances, the T2 value of IO-DyO NPs in the liver decreases significantly, and the diagnostic sensitivity is higher at 9.4 T than at 7.0 T (Fig. S4E). The IO-DyO NP contrast agent expands the range of materials available for enhancing image contrast in ultrahigh-field MRI. Furthermore, these materials can be explored for their therapeutic potential. By leveraging the inherent properties of Fe and Dy iron, IO-DyO NPs demonstrate photothermal capability under the influence of an external alternating magnetic field (Fe) and up-conversion property (Dy). This presents exciting opportunities for image-guided therapy in liver-related diseases.

Molecular MRI, through the utilization of USPIO, modified with RGD peptide to specifically target integrin α_v_β_3_, has demonstrated remarkable efficacy in the early detection of tumor angiogenesis. Additionally, this technique has been proven effective in monitoring tumor responses to antiangiogenic treatments in xenograft models [165]. Zhang et al. [166] have shown for the first time that molecular MRI can assess liver fibrosis in a CCl_4_ rat model using an RGD peptide-modified USPIO (RGD-USPIO) probe that targets integrin α_v_β_3_. The changes in transverse relaxation rate (ΔR2) of RGD-USPIO and unmodified USPIO were compared in MRI. The results showed that ΔR2 values have significant differences at various stages of liver fibrosis after RGD-USPIO injection compared to unmodified USPIO.

Furthermore, in fibrotic liver specimens, the accumulation of iron particles was significantly higher with RGD-USPIO than naked USPIO. It was mainly taken up by aHSCs rather than the resident macrophages of liver. Thus, the use of RGD-USPIO conjugated to integrin α_v_β_3_ expressed on aHSCs could be sensitive enough to detect sparse targets of early liver fibrosis in vivo (137.85 mM s^−1^ at 1.41 T MR) and to distinguish between different stages of liver fibrosis [166].

#### Mn-based contrast agents

Mn^2+^ is an endogenous ion in the body and an essential component of normal cellular function. It often serves as a cofactor for enzymes and receptors [167]. At the biochemical level, Mn is involved in mitochondrial function, and its absorption level correlates positively with the mitochondria density in cells. Since hepatocytes are abundant in mitochondria, and Mn^2+^ is a paramagnetic metal ion capable of providing significant MRI contrast, Mn is an excellent contrast agent for liver MRI. It shortens the longitudinal relaxation time T1 of water protons through dipolar interaction with the unpaired electrons of metal ion [148,168]. Although free Mn^2+^ is less toxic than free Gd^3+^, and its intravenous administration benefits from biliary excretion, particularly when renal function is compromised [169], high concentrations of Mn^2+^can be neurotoxic and can interfere with myocardial Ca^2+^ processing, potentially leading to cardiovascular toxicity. Therefore, to safely use Mn^2+^ as intravenous contrast agents in humans, it must be sequestered to mitigate the risk of free metal-based toxicities [161,170].

Recently, studies on Mn chelates and Mn NPs as potential MRI contrast agents have been developed. Even small-molecule chelated contrast agents have been classically entrapped into liposomes as NPs [168,170]. Mn chelates, such as mangafodipir trisodium (Mn-DPDP), act as contrast agents that target the hepatobiliary system, being selectively taken up by functional hepatocytes and eliminated through the bile. By their paramagnetic properties, Mn-DPDP enhances the brightness of T1-weighted images, resulting in improved visualization of the liver and biliary tree [171]. Previously, it was the only Mn-based contrast agent approved by the FDA. However, given that any hepatocellular tissue, including lesions, is enhanced by nonspecific Mn^2+^ in a similar time course, Mn-DPDP-enhanced MRI usually fails to significantly distinguish lesions in the liver [161]. Ning et al. [172] introduced 2 piperazino-hydrazine into the backbone of cis-Mn-1,4-DO2A, forming Mn(II) complexes named Mn-2CHyd. Mn-2Hyd and Mn-1CHyd were additionally synthesized as controls (Fig. S5A). Mn-2CHyd, a novel hydrazine-coordinated Mn-based MRI probe, has higher affinity and turn-on relaxivity (4-fold) than the single-binder method by covalently reacting with Lys^Ald^ for detection, staging, and quantification of liver fibrogenesis (Fig. S5B). Mn-2Hyd, which was radiolabeled with the positron-emitting isotope ^52^Mn, has a lower nonspecific liver background signal than Mn-1CHyd measured by PET-MRI in normal mice, and both probes showed rapid elimination through the kidneys (Fig. S5C and D). In CCl_4_-treated mice, the enhancement of the liver and ∆CNR by Mn-2CHyd was significantly higher than that of Gd-DOTA. Compared to the group treated with olive oil vehicle, CCl_4_-treated mice showed a slower liver clearance (Fig. S5E and F). Mn-2CHyd shows the effectiveness of the dual binding approach by restricting molecular rotation, which enhances relaxivity upon binding and has potential to target Lys^Ald^ for detecting liver fibrogenesis. Therefore, this PET-MRI dual-modality probe could be used for future accurate and quantitative assessments of liver fibrosis.

### PET

PET is a molecular imaging technology that employs radiolabeled tracers incorporating radionuclides. The radionuclide attached to the probe molecule’s atoms releases a positron during positron decay, which travels a short distance before annihilating with an electron in the surrounding substance, resulting in the production of two 511-keV γ-rays emitting simultaneously in opposite directions. Then, the surrounding detectors detect the γ-rays and form an image through reconstruction. This method provides a sensitive means for early identification and characterization of disease properties [173,174]. The unique advantages of PET are reflected in the following aspects: First, the radionuclides used for labeling may include isotopes of carbon, nitrogen, and oxygen components of biological macromolecules. These isotopes do not alter the physicochemical and biochemical properties of the imaging agent. Additionally, although fluorine atoms are not biomolecular components, they can replace hydrogen atoms or hydroxyl groups as the most commonly used bioisosteric replacements [175]. Second, as a radionuclide-based imaging modality, PET has the capability to determine the concentration of specific biomolecules down to the picomolar range. Thus, PET can detect biological abnormalities sensitively before changes manifest, aiding in identifying early-stage diseases, distinguishing between benign and malignant lesions, and determining therapeutic effectiveness. It has a diagnostic accuracy of 8 to 43% higher than traditional methods and allows for treatment plan adjustments based on clinical scenarios. Furthermore, the synthesis of PET probes involves very low mass, which helps to avoid any mass-related impact on the measured biological processes [176].

PET also has some limitations; despite being highly quantitative, it lacks spatial resolution and sufficient anatomical information. To address this, PET/CT is used to add anatomical details. However, due to the ionizing radiation it emits, there has been a push toward the development of PET/MRI. This emerging technique combines PET and MRI, taking advantage of the inherent benefits of MRI, such as enhanced soft tissue contrast and reduced radiation exposure. PET/MRI provides both anatomical and functional information, which makes it a promising alternative in clinical settings [177].

On the one hand, the development of new PET probes for liver fibrosis imaging can utilize nuclides with relatively long half-life, such as ^18^F and ^64^Cu, to label radiotracers, thereby extending the imaging time window [178]. On the other hand, radionuclides primarily excreted through nonhepatic pathways, such as ^18^F and ^68^Ga, can be selected to minimize background uptake and dosimetry to improve the imaging quality [178]. Given that ^18^F has a lower positron energy and a physical half-life of 110 min, it enables more complex radiosynthesis and extended in vivo studies. Consequently, the majority of current contrast agents used for PET imaging of liver fibrosis are ^18^F-labeled radiopharmaceuticals [175]. 2-Deoxy-2-[^18^F]fluoro-D-glucose ([^18^F]FDG) is the most widely recognized commercial PET radiopharmaceutical, frequently used for imaging tumors and increasingly utilized for visualizing infectious, inflammatory, and degenerative diseases [179].

Recent studies have shown that [^18^F]FDG PET/CT can quantitatively assess liver metabolism in patients at different stages of liver fibrosis/cirrhosis [180]. Hatori et al. [181] developed *N*-benzyl-*N*-methyl-2-[7,8-dihydro-7-(2-[^18^F]fluoroethyl)-8-oxo-2-phenyl-*9H*-purin-9-yl]acetamide ([^18^F]FEDAC), which is a radioprobe specific for TSPO expressed by macrophages and aHSCs, for the noninvasive visualization of liver fibrosis in vivo using PET. [^18^F]FEDAC has also been employed for visualizing NAFLD noninvasively [123]. In a rat model of liver fibrosis, the accumulation and heterogeneous distribution of radioactivity from [^18^F]FEDAC increased with both the duration of CCl_4_ treatment and the severity of liver damage compared to the control group (Fig. S6A). The expression of TSPO by macrophages and aHSCs increased progressively. In vitro studies using radiographic autoradiography revealed a strong correlation between the radioactivity distribution derived from [^18^F]FEDAC and TSPO expression (Fig. S6B). Thus, PET imaging with the TSPO-specific radioprobe [^18^F]FEDAC may aid in the noninvasive visualization of liver fibrosis progression to cirrhosis in patients with chronic liver diseases [181].

A potential drawback of PET technology is the requirement for an expensive infrastructure, such as a cyclotron, for on-site radionuclide production. In contrast, radiometals like ^68^Ga can be produced by generators instead of cyclotrons, which makes them increasingly favored for diagnosing liver fibrosis [182]. Rosestedt et al. [183] developed a potential PET tracer for liver fibrosis detection, called [^68^Ga]Ga-DOTA-PEG_2_-LRELHLNNN. This tracer was designed to selectively target type I collagen, which is a major component of fibrotic liver tissue. [^68^Ga]Ga-DOTA-PEG_2_-LRELHLNNN exhibited a higher binding to fibrotic liver tissue than to the surrounding tissues in mice. However, [^68^Ga]Ga-DOTA-PEG_2_-LRELHLNNN was observed to undergo in vivo protein degradation, which limited its targeting specificity to the liver. Velikyan et al. [184] developed a novel radiolabeled collagelin analog that targets type I collagen, [^68^Ga]Ga-NO2A-[Nle^13^]-Col. This compound was modified by substituting methionine for a norleucine amino acid, which enhanced peptide stability against radiation degradation, achieved high radiochemical purity, and maintained consistent organ distribution. Autoradiography studies of frozen liver tissue sections showed a higher uptake in fibrotic tissue compared to normal tissue. Additionally, a linear correlation was found between tracer uptake and the severity of fibrosis, as determined by the fibrosis scoring (Fig. S6C and D).

Furthermore, [^68^Ga]Ga-NO2A-[Nle^13^]-Col has shown rapid blood clearance and tissue washout in both healthy and diseased mice. This characteristic contributes to a favorable radiation dosimetry profile, which is considered safe and permits repeated scanning. In conclusion, [^68^Ga]Ga-NO2A-[Nle^13^]-Col exhibits promise for in vivo applications, including fibrosis staging, monitoring disease progression, and evaluating treatment response [184].

### SPECT

SPECT is a sensitive nuclear imaging technique that offers an in vivo 3-dimensional (3D) spatial distribution of radionuclides emitting single photons [185]. Heavy radioisotopes in the body emit γ photons through radioactive decay, and the γ camera in SPECT rotates around the subject, which detects γ photons from multiple positions during the rotation to acquire projections and subsequently reconstruct SPECT images [186]. SPECT and PET share similarities in their utilization of radiotracers and detection of γ-rays. However, significant differences do exist. First, SPECT employs radioisotopes that emit only one γ-ray during decay, and these are directly measured. Second, the collimator in the γ camera serves as a filter, absorbing most photons, resulting in significantly lower sensitivity than PET. Despite this, SPECT is more commonly used than PET for several reasons: SPECT scans are more affordable, the half-life of SPECT nuclides is longer, and they are more accessible [186,187]. Technetium-99m (^99m^Tc) is one of the most commonly used radioisotopes in SPECT, emitting 140-keV γ-rays with a half-life of 6.006 h, sufficient for radiolabeling and imaging procedures and adequately brief to minimize unnecessary radiation exposure to patients [188]. In addition to ^99m^Tc, other commonly used radionuclides in SPECT include gallium-67 (^67^Ga), iodine-123 (^123^I), and indium-111 (^111^In). These radionuclides possess unique spectra, which facilitate the use of multiple probes labeled with different radionuclides. This capability enables the simultaneous investigation of various cellular or molecular events in SPECT imaging [185].

However, SPECT suffers from low spatial resolution and provides limited anatomical information. The dual-modality system, which combines SPECT and CT, facilitates the simultaneous acquisition of functional and detailed anatomical information. This integration allows for precise localization and quantification of labeled imaging probes, thus enhancing the accuracy of diagnostic imaging [189]. In the past few years, significant advances have occurred in the development of SPECT radiotracers for noninvasive imaging of liver fibrosis, including peptides, proteins, antibodies, and more. There is an urgent need to develop novel, specific, and sensitive SPECT imaging agents to detect liver fibrosis [190]. Yu et al. [191] developed an enhanced dimeric RGD radiotracer, termed technetium 99m-PEG_4_-E[PEG_4_-cyclo(arginine-glycine-aspartic acid-d-phenylalanine-lysine)]_2_ (^99m^Tc-3PRGD2). This radiotracer exhibited a significantly higher affinity to integrin α_v_β_3_ expressed by aHSCs than the monomeric RGD radiotracers. The SPECT/CT image signal of ^99m^Tc-3PRGD2 exceeded that of the control group as early as 4 weeks in mice with liver fibrosis, and the tracer’s accumulation in the liver escalated with the progression of liver fibrosis (Fig. S6E). The SPECT/CT quantification accuracy was further validated through in vitro biodistribution analysis, demonstrating a linear relationship in the radiotracer distribution (Fig. S6F). Following treatment with PEGylated interferon-a2b (IFN-a2b), the liver tissue uptake of ^99m^Tc-3PRGD2 was notably reduced compared to the control or spontaneous recovery groups. This finding underscores the potential of ^99m^Tc-3PRGD2 as a valuable tool for SPECT/CT imaging. This radiotracer can effectively monitor the progression and recovery of liver fibrosis by providing both structural and functional imaging details. Furthermore, it shows promise as a noninvasive and early diagnostic method for liver fibrosis.

### FI

FI is an imaging technique in which an appropriate light source excites a fluorescent contrast agent, which then emits light that is captured by a specialized camera to form an image. It offers sufficient sensitivity and contrast to visualize the organ or tissue of interest [192]. Compared to MRI, CT, and PET, FI provides advantages such as nonionizing radiation, high spatial resolution, portability, and affordability. In addition, it can provide real-time imaging of whole organs at the macroscopic scale [193]. In the visible window, light that is irradiated to tissue is scattered and absorbed by molecules like hemoglobin, leading to a significant decrease in tissue light signals. The introduction of the first near-infrared window (NIR-I, 750 to 900 nm) has revolutionized in vivo FI by providing a deeper tissue penetration (1 to 3.5 mm) [194].

Nevertheless, the limited imaging depth continues to pose a challenge in NIR-I FI [194]. The demand for more accurate imaging in complex pathophysiological conditions has driven the need for FI with improved depth penetration and enhanced signal-to-background ratio (SBR). The second near-infrared window (NIR-II), which has a longer wavelength, comprises two subwindows: NIR-IIa (1,300 to 1,400 nm) and NIR-IIb (1,500 to 1,700 nm). FI in the NIR-IIa/IIb windows yields reduced photon scattering and substantially attenuated autofluorescence levels in the NIR-IIb window. Merging these benefits, NIR-IIa/IIb imaging facilitates not merely high-resolution but also deep-tissue imaging [194–196].

In current clinical practice, 2 small-molecule NIR dyes are utilized: indocyanine green (ICG) and methylene blue. ICG is the only approved small-molecule NIR fluorescent contrast agent for surgical applications, emitting at approximately 800 nm. Being exclusively metabolized by the liver, ICG is useful for assessing liver clearance capacity and hepatic lesions. However, ICG and methylene blue, as nontargeted small-molecule contrast agents, are rapidly cleared and they generate high background signals, thereby reducing the quality of FI [197].

As a means to improve imaging quality, there has been a shift toward molecular FI with the employment of more specific fluorescent agents, departing from the traditional approach of structural and functional FI [192]. Fluorescent probes with high targeting, high brightness, optimal biocompatibility, and excellent pharmacokinetics are being progressively developed for liver fibrosis imaging [196]. Yang et al. [198] developed a mitochondrial-targeted NIR fluorescence probe called DHMP2 for detecting MAO-A, which acts as a target in liver fibrosis diagnosis. DHMP2 exhibits strong NIR emission (>700 nm), which could offer good tissue penetration and minimal photo damage in vivo, as compared to visible light. The experimental results showed that upon injecting DHMP2 into both normal and CCl_4_-induced liver fibrosis rats, bright NIR fluorescence signals were observed. Conversely, liver tissues treated with clorgyline, a specific inhibitor of MAO-A, exhibited weak fluorescence signals. In this study, DHMP2, as the first NIR fluorescence probe, demonstrated good capability in real-time visualization of MAO-A activity in rat liver fibrosis. The results showed that DHMP2 exhibits high sensitivity, good selectivity, and important safety, and thus holds good promise for in vivo monitoring of MAO-A activity and early diagnosis of liver fibrosis.

Ding et al. [199] utilized CH1055, a small-molecule dye platform known for its exceptional biocompatible and easy functionalization, as an NIR-II organic dye. They integrated it with various short PEG linkers to regulate the self-assembly of the probe, culminating in the creation of a series of NIR-II probes designated as SCH1 to SCH4 (Fig. S7A). Among them, the single molecular probe SCH4 demonstrates excellent photostability and urinary excretion rate, with an emission peak at 1,050 nm. This probe has been leveraged for NIR-II imaging of liver fibrosis in mice. In normal mice liver, fluorescent signals were observed, while fibrotic mice exhibit nondetectable fluorescent signals (Fig. S7B). The hypothesis suggests that hepatic fibrosis triggers the persistent growth of connective tissue, replacing the normal liver function and causing a decrease in portal blood flow. As compared to ICG, SCH4 as an NIR-II probe has lower scattering and deeper penetration in tissues. This enables the SCH4 probe to more easily identify early-stage liver fibrosis in vivo.

Recently, Jiang et al. [200] developed an intelligent nanoplatform termed LnNRs@mSiO_2_-RBS for NIR-II ratiometric imaging. This platform combines lanthanide nanorods with photo-triggered NO releasing molecules, enabling the concurrent diagnosis and therapeutic treatment of liver fibrosis. Under 808-nm laser irradiation, liver fibrotic mice exhibited NIR-IIa emission (1,000 to 1,400 nm) from innate lipofuscin pigments in the fibrotic region, facilitating label-free early detection of liver fibrosis. Under 980-nm laser excitation, this nanoplatform not only released NO as required for fibrosis therapy but also emitted light at 1,532 nm. Notably, after NO gas therapy, the intensity of the NIR-IIa emission from the inherent pigment sharply declined, whereas the NIR-IIb signal (1,532 nm) escalated. They together serve as a self-calibration reference indicator for ratiometric imaging (*I*_980 nm Ex_/*I*_808 nm Ex_), allowing for monitoring of the NO gas therapy procedure. In conclusion, this novel nanoplatform offers an innovative strategy for diagnosing and treating liver fibrosis. Moreover, it provides guidance to clinicians, predicts treatment outcomes, and reduces risks, making it highly promising for clinical applications.

Although, there are still relatively few reports on NIR-II molecular probe-based FI of liver fibrosis, this technology harbors enormous potential and represents a promising direction for future development.

### PAI

PAI operates based on the principle of the photoacoustic effect. When an object is exposed to electromagnetic radiation, a portion of the energy is absorbed and converted into heat. This thermal energy causes the object to expand, thereby generating acoustic pressure waves. These pressure waves, in the form of ultrasonic waves, propagate through the medium and can be detected using ultrasonic sensors for subsequent imaging purposes [201]. PAI is a rapidly emerging biomedical technique that effectively combines the strengths of optical imaging, offering high absorption contrast, with US, which provides high spatial resolution. By utilizing this hybrid approach, PAI facilitates the acquisition of detailed structural, functional, and molecular imaging capabilities. This integration of modalities has positioned PAI as one of the fastest-growing and most promising methods in the field of biomedical imaging [202,203]. Contrast agents with high light absorbance in the NIR spectral region, such as small molecules (e.g., dyes) and NPs, have enhanced the sensitivity and specificity of PAI [204].

PAI contrast agents with high absorbance in the NIR spectral region are particularly effective for detecting deeper tissue. A typical example is ICG, which is known to significantly enhance PAI signal when injected into blood vessels [205]. Lv et al. [206] employed dynamic contrast-enhanced PAI (DCE PAI) to study the pharmacokinetics of ICG in liver fibrosis mice. DCE PAI revealed that the dynamic parameters [maximum peak time (*T*_max_) and half-life (*T*_1/2_)] of disease models were longer than those in control mice without fibrosis and differed among model groups (Fig. S7C to E). *T*_max_ and *T*_1/2_ were positively correlated with histologic fibrosis measures, indicating that the extent of liver fibrosis can be assessed by ICG metabolism. DCE PAI utilizes this information to enable early diagnosis and staging of liver fibrosis. By analyzing ICG uptake and clearance kinetics, DCE PAI offers a noninvasive approach for early-stage liver fibrosis assessment. However, small-molecule dyes are generally not suitable for long-term monitoring in PAI due to their short cycle time. In contrast, NPs feature a tunable absorption spectrum and a longer circulation time. By adjusting the shape and size of NPs, their absorption peaks can be tailored to the desired imaging wavelength. Moreover, NPs can be specifically targeted to sites by coupling them with ligands like antibodies. This targeted approach significantly improves the accuracy and specificity of PAI, making NPs invaluable for long-term monitoring and molecular imaging [207]. NPs for PAI in liver fibrosis have been scarcely reported but represent the future direction for realizing targeted PAI.

### US

As a clinical imaging modality, US provides real-time, high-resolution, and morphological imaging (using harmonics). Conventional US presents advantages such as cost-effectiveness, portability, and the absence of ionizing radiation, which are also applicable for US-based elastography [208]. Currently, US-based elastographies, like vibration-controlled transient elastography (VCTE or Fibroscan), point sheer wave elastography [pSWE or acoustic radiation force impulse (ARFI)], and 2-dimensional SWE (2D-SWE), have achieved remarkable breakthroughs in the noninvasively qualitative and quantitative assessment of liver fibrosis by measuring liver stiffness [209]. For VCTE, the widely used and validated technique for identifying advanced fibrosis and cirrhosis, it measures the velocity of a shear wave produced mechanically across the liver [210]. As to SWE, it utilizes acoustic radiation force to produce tissue displacement, resulting in the formation and propagation of perpendicular shear waves. While pSWE applies forces at a single location, 2D-SWE applies forces at multiple locations to generate a 2D elastographic tissue map [211]. These techniques are primarily recommended for distinguishing between significant or advanced fibrosis and nonsignificant fibrosis due to their current limitations in accurately differentiating between different stages of fibrosis [212].

It is envisioned that US molecular imaging presents a more appealing strategy for early diagnosis of liver fibrosis. To perform US molecular imaging, it is essential to utilize a highly effective contrast agent capable of efficiently backscattering US waves. This plays a crucial role in detecting lesions and differentiating them from surrounding tissues. Consequently, a variety of US contrast agents (UCAs) have been developed, including nanobubbles, MBs, echogenic liposomes, and phase change contrast agents. Among them, MBs are the most commonly employed UCAs. MBs comprise a biopolymer, amphiphilic lipid, or albumin shell surrounding a gas core. With a size range of 1 to 8 μm, MBs can transport unhindered within the microcirculation [213]. However, due to their size, MBs and most US NPs are confined to the intravascular space. They function purely as blood pool contrast agent, capable only of imaging molecular targets present in the bloodstream or on the surface of vascular endothelial cells.

During the development of liver fibrosis, there is a significant increase in angiogenesis and neovascularization, leading to enhanced vascular permeability [214]. Assuming that the US NPs are sufficiently small to cross the endothelial gap of neovascularization (at the nanoscale), they can attach targeting ligands for extended retention in the ECM. Such NPs could serve as molecular imaging agents, selectively accumulating and imaging molecular targets outside the vasculature [215]. Xuan et al. [216] covalently modified cRGD octapeptide onto Poly (lactic-co-glycolic acid) (PLGA) NPs encapsulating perfluorooctyl bromide (PFOB), resulting in the synthesis of cRGD-PLGA-PFOB core-shell NPs that can serve as UCAs. This promoted specific binding to the integrin α_v_β_3_ expressed on aHSCs during liver fibrosis. In this process, PFOB liquid serves as the core to provide echogenicity, while organic PLGA NPs functioned as the preferred targeting carrier material with good stability and extended in vivo circulation time (Fig. S8A). Scanning electron microscopy (SEM) images revealed the NPs’ predominantly spherical shape and smooth surfaces (Fig. S8B). In vivo, high-frequency US imaging of rats with liver fibrosis demonstrated a noteworthy increase in echo intensity within the livers of rats injected with cRGD-PLGA-PFOB NPs, compared to pre-injection levels or rats injected with PLGA-PFOB NPs. Additionally, a positive correlation was observed between the intensity of the echo signal and the degree of liver fibrosis (Fig. S8C). Therefore, cRGD-PLGA-PFOB NPs serve as an effective targeted probe for US molecular imaging, offering a straightforward, noninvasive, and precise method for dynamic monitoring and differentiation of liver fibrosis stages.

### Raman imaging

Raman spectroscopy is a technique for characterizing molecules by measuring inelastic light-scattering effects [208]. Spontaneous Raman spectroscopy frequently employs NIR light for probing deep tissues due to its minimal laser damage and excitation of autofluorescence. However, the signal generated by spontaneous Raman scattering is weak, which has prompted the development of multiple enhancement techniques to tackle this issue [217]. Among these techniques, surface-enhanced Raman scattering (SERS) is the most commonly utilized. Surface plasmon resonance (SPR) occurs when an analyte molecule directly interacts with a metal surface. Suppose that the incident light’s frequency corresponds to the inherent oscillation frequency of unbound electrons within the metal. In metal nanostructures like gold, silver, and copper, SPR can exhibit significant localization at specific sites [218,219]. Incident light can be effectively coupled into metal NPs, increasing the local electromagnetic field intensity on the surface of NPs by 2 to 5 orders of magnitude [219]. When molecules are positioned near plasmonic materials, they can generate Raman scattering signals with a strength increased by 10^4^ to 10^6^ times. In summary, SERS can amplify the electromagnetic fields generated by the excitation of localized surface plasmons, enabling accurate and sensitive structural detection of low concentration analytes [220].

The SERS targeting probe that can accurately diagnose early liver fibrosis in vivo has been rarely reported. Xiang et al. [221] synthesized gold nanostar (GNS) liver-targeting tags (GLTTs) by modifying the coupling of glycyrrhetinic acid (GA) and functionalized PEG onto the surface of GNSs. This was done for the SERS-based detection of liver fibrosis, where GNSs served as SERS materials and the tip structure “hot spots” on their surface yielded exceptionally high SERS enhancement [219], and GA specifically binds to hepatic parenchymal cells (Fig. S8D). GLTTs have the advantages of good biocompatibility, high stability, and low toxicity. GLTTs and nontargeting GNSs were respectively administered to fibrotic and normal mice, and SERS spectra were subsequently collected. The results showed that GLTTs were 12.85-fold more effective at detecting liver tissue than GNSs (Fig. S8E), and the assay exhibited improved reproducibility as well. Consequently, it is anticipated that GLTTs could identify the metabolism of characteristic peaks (lipid, glucose, amino acid and protein) via SERS spectra to detect early liver fibrosis.

In summary, each imaging modalities have their own pros and cons for liver fibrosis diagnosis in vivo. For MRI, it is a common noninvasive imaging technique with deep penetration, which is suitable for clinic diagnosis of liver fibrosis. Importantly, with the development of modern MRI technology and AI, it could provide high spatial resolution to submillimeter, good soft tissue contrast, and even anatomical information in 3D manner. With the assistance of targeted molecular contrast agents, MRI is helpful to pinpoint the margin of liver fibrosis tissues in early stage. It is noted that generally MRI requires rather long processing time to collect sufficient signals, and high local concentration of contrast agents in disease lesion, while it is also quite challenging so far to collect whole-body images shortly because of the limited operation dimension for the MRI instrument [138]. For PET and SPECT, they have intrinsically high sensitivity and could assist timely and quantitative imaging of molecular processes involving fibrosis with radiolabeled trackers. It should be borne in mind that PET and SPECT exhibit ionizing radiation, low spatial resolution, and lack of anatomical information. As compared to SPECT, PET offers greater sensitivity and utilizes more durable and adaptable tracers, but the high cost of cyclotron of PET limits its accessibility to wide patients [182,222]. As to FI, it has merits including high spatiotemporal resolution, noninvasive detection, real-time monitoring capabilities, and ease in operation. Generally, it is limited by the tissue penetration depth, which restricts its application in diagnosing liver fibrosis beneath skins. As compared to conventional visible/NIR-I FI, the emerging NIR-II imaging offers enhanced spatial resolution, improved imaging depth sensitivity, and increased imaging contrast, because of decreased photon scattering, reduced tissue autofluorescence, and lowered photon absorption at longer wavelengths. However, the absence of suitable imaging instruments and optical probes has prevented the evaluation of NIR-II imaging in clinical settings [223]. For PAI, it offers fast imaging, deeper tissue penetration, scalable high spatial resolution, and safe operation, but it faces challenges in quantitative analysis [224]. For US, it is a primary imaging tool for suspected liver fibrosis patients, offering real-time imaging, simplicity, affordability, repeatability, noninvasiveness, and wide accessibility, without ionizing radiation. However, its diagnostic ability is limited due to significant interobserver variability and low specificity [225]. For Raman imaging, it possesses advantages such as real-time capability, high sensitivity, shorter spectrum bandwidth, the ability to utilize stable molecular probes, and a higher signal-to-noise ratio. However, it also suffers from the limitation of low penetration of light into tissues [226].

Multimodal imaging combines different imaging modes to provide more precise and detailed information as compared to single imaging modalities. For instance, commercial PET/CT or PET/MR systems merge the high-sensitivity functional data from PET with the high-resolution anatomical data from CT and MRI. Importantly, multimodal imaging probes have been developed to carry multiple imaging agents, offering detailed target-specific information through targeted delivery. For example, Miao et al. [227] developed an activatable fluoro-photoacoustic dual-modal imaging probe termed IP for noninvasive detection of early-stage liver fibrosis, which specifically recognizes integrin through cRGD. Therefore, development of contrast agents with multimodal imaging functionality is promising for precise diagnosis of liver fibrosis [228,229].

In recent years, AI algorithms, especially deep learning, have demonstrated value in clinic diagnosis with integration of conventional imaging modalities especially for automatic quantification of medical imaging data. They simulate human intelligence’s learning, classification, problem-solving, and decision-making capabilities through computer programs [230]. The development of AI has also promoted noninvasive applications for liver disease prediction and diagnosis. For example, a recent study has demonstrated that a deep learning model based on hepatobiliary phase gadoxetic acid-enhanced MRI exhibits excellent performance in the staging diagnosis of liver fibrosis, with diagnostic efficacy comparable to MRE [231]. AI as an auxiliary tool in the clinical workflow is of great help to enhance clinicians’ expertise and decision-making abilities, reduce misdiagnosis rates, and facilitate early intervention and optimized clinical care. However, AI also faces challenges, such as dependence on high-quality data, poor model interpretability, and data security issues. Nevertheless, these challenges are being continuously improved, indicating a broad future prospect for AI in the medical field [232].

## Molecular Imaging Probes in Clinical Practice

Currently, there are few molecular imaging probes used in clinical practice or clinical trials for liver fibrosis. Most of the molecular imaging agents are still in the laboratory research stage. For instance, SPIOs are particles formed by small crystals of iron oxide (typically magnetite Fe_3_O_4_ or γ-Fe_2_O_3_) with surfactant coating, which have been successfully used for detecting liver diseases, including liver fibrosis [162]. So far, several SPIOs, such as Feridex, Resovist, and Combidex, have been developed and clinically tested as MRI contrast agents in clinic. However, currently, Resovist is the only one available for clinic usage in certain countries, while other SPIOs have been discontinued or withdrawn from the market. Additionally, Feraheme, another SPIO agent, has received approval for treating iron deficiency in adult patients with chronic kidney disease, and its imaging potential is still under research [233].

In addition, gadolinium ethoxybenzyl-diethylenetriaminepentaacetic acid (Gd-EOB-DTPA) is an approved hepatocyte-specific MRI contrast agent, which is extensively employed for detection and characterization of liver tumors. This agent is absorbed by liver cells via the organ-anion transporters (OATPB1/B3) found in sinusoids and subsequently excreted into the bile ducts mediated by the adenosine triphosphate (ATP)-dependent multidrug resistance protein 2 (MRP2) [234]. About half of the Gd-EOB-DTPA is ejected via the biliary system, while the kidneys eliminate the remaining 50%. The uptake of Gd-EOB-DTPA by the liver depends on the integrity of hepatocyte mass. As liver fibrosis advances, alterations in hepatic hemodynamics and functional impairment lead to a significant decrease in Gd-EOB-DTPA uptake in the liver parenchyma. This feature contributes to the diagnosis and distinction of different stages of liver fibrosis [235].

Despite some progress in the field of molecular imaging for liver fibrosis, targeted molecular imaging probes still remains in the research stage. Further laboratory research and clinical trials are essential to confirm their efficiency and safety in diagnosing liver fibrosis, with the aim of providing more precise and reliable diagnostic outcomes for liver fibrosis.

## Challenges and Outlook

To date, considerable progress has been made in studying the cellular and molecular mechanisms of liver fibrosis. The study of varied biomarkers’ expression has further propelled the development of liver-targeted molecular imaging. In animals, most of the specific molecular imaging probes for liver fibrosis, particularly those targeting aHSCs, macrophages, and collagen, have been implemented. However, no targeted molecular contrast agents for liver fibrosis are currently available for human use. A multitude of published researches suggest that the molecular contrast agents developed for liver fibrosis hold great promise for future clinical applications. The first challenge lies in the fact that, although most molecular contrast agents have demonstrated good biocompatibility in vitro and in vivo experiments, their long-term safety remains unverified. For instance, chronic liver toxicity can result from the prolonged use of NPs, attributable to their slow clearance and retention in the body. Furthermore, patients with liver disease are more susceptible to the toxicity of probes. The following strategies can be employed to enhance the safety of molecular contrast agents for liver fibrosis: (a) Systematic evaluation of the immunogenicity, long-term toxicity, and pharmacokinetics of molecular probes. (b) Formulation of probes with optimal size, composition, and surface chemistry to establish equilibrium between prolonged in vivo circulation and probe safety [41]. (c) Development of molecular probes that specifically target liver fibrosis and are primarily excreted through nonhepatic pathways, such as renal clearance. This approach can reduce the injection dose and background uptake necessary for imaging, aiding in the development of molecular probes suitable for patients with liver disease [178]. The second challenge is the potential discrepancy in the concentration of liver fibrosis imaging targets between animal models and human liver fibrosis. Variances in the pharmacokinetics and metabolism of probes in humans necessitate further scrutiny of these probes’ performance in nonhuman primate models. The third challenge emerges from the typically prolonged duration of fibrosis progression and regression studies, rendering clinical trials frequently complex and expensive [236]. However, given that liver fibrosis is a common characteristic of many chronic diseases, molecular imaging probes for liver fibrosis can also be broadly applicable to numerous other diseases associated with fibrosis. Consequently, the potential market size could be considerable, generating a high return on investment [42,67].

Efforts are being made to develop a unified platform, known as “theranostics”, that integrates diagnostic and therapeutic functions [237]. The imaging probes targeted for identifying liver fibrosis, along with the therapeutic agents developed for its treatment, aim to visually deliver/release drugs into the organism, provide precise, personalized drug regimens, and monitor treatment effects, allowing for timely adjustments to the patient’s treatment plan. For example, Wu et al. [238] synthesized a pH-sensitive VA-linked copolymer, T-PBP, and assembled it into SPIO-decorated cationic micelles. T-PBP micelles efficiently transported miRNA-29b and miRNA-122 to HSCs in an MRI-visible manner, producing synergistic anti-fibrotic therapeutic effects by down-regulating fibrosis-related gene expression. Kim et al. [239] synthesized a PEI-D-GlcNAc-ICG/TGFb1siRNA complex, aiming at aHSCs through the specific binding of GlcNAc to vimentin and desmin. FI of ICG facilitates liver fibrosis diagnosis, and TGFb1siRNA attenuates inflammation levels in liver fibrosis. Thus, the PEI-D-GlcNAc-ICG-TGFb1siRNA complex can simultaneously analyze and treat liver fibrosis. Hu et al. [240] developed a novel NP composed of iron oxide (Fe_3_O_4_) NPs (IONPs) and ferulic acid (FA) co-encapsulated with PLGA NPs. The surface of the NPs were modified with cRGD peptides (referred to as cRGD-PLGA/IOFA) for targeted delivery to integrin α_v_β_3_ in liver fibrosis. The encapsulated FA and IONPs in cRGD-PLGA/IOFA effectively alleviate liver fibrosis by acting on HSCs and macrophages, resulting in a synergistic collagen degradation effect and enhanced therapeutic efficacy. Therefore, this innovative approach provides a new avenue for clinical MRI-guided treatment of liver fibrosis. Image-guided therapies for liver fibrosis fully exploit the synergy between diagnosis and treatment, leaning on the advancements in molecular imaging technology. This technology allows therapeutic drugs to have a more significant impact and pave the way for more efficient personalized medicine.

## Conclusion

At present, there are a few available protocols in the clinic for the treatment of liver fibrosis in early stage, which is mainly due to the lack of noninvasive diagnosis and longitudinal evaluation paradigms. The molecular imaging technologies are promising to revolutionize the diagnosis, staging, and efficacy monitoring of liver fibrosis in clinic, which is thus summarized in this review. The efforts for development of molecular imaging for precise imaging of liver fibrosis in early stage will expedite the translation of imaging probes from bench to bedside, which is also of significant importance for innovating the therapeutic paradigms, and ultimately improving prognosis of patient-bearing liver fibrosis.
