# Supplementary material for: Advances in Noninvasive Molecular Imaging Probes for Liver Fibrosis Diagnosis
Source: Biomater Res. 2024 Jul 1;28:0042. doi: 10.34133/bmr.0042 (PMC11214848; doi:10.34133/bmr.0042)
Supplement: Supplementary 1 — Figs. S1 to S8 [file bmr.0042.f1.docx]

**Supplementary material**

**Supplementary Fig. 1**


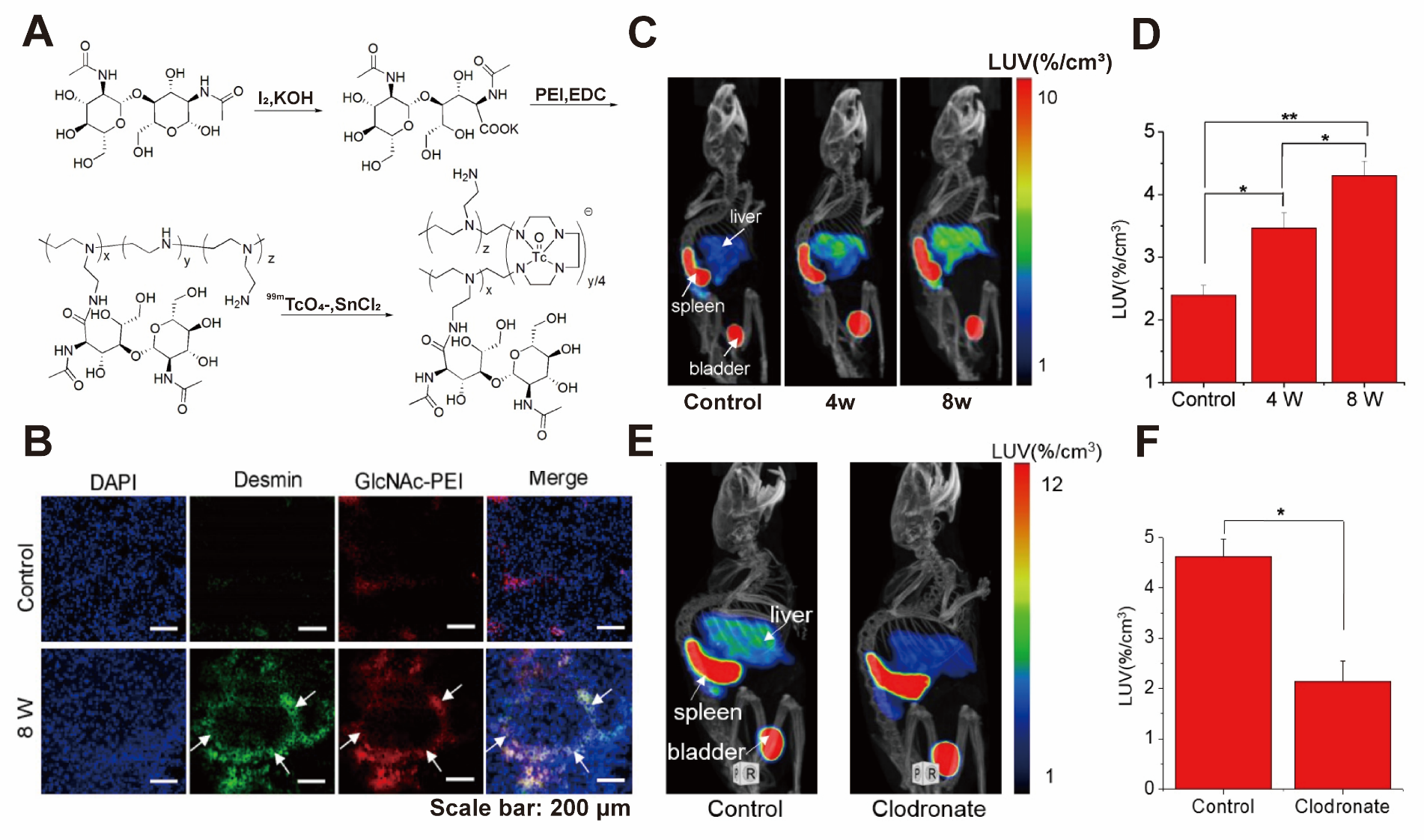


**Supplementary Fig. 1** Targeting Desmin and vimentin on aHSCs for imaging liver fibrosis. **(A)** Synthesis and radiolabeling of ^99m^Tc-GlcNAc-PEI. **(B)** Immunofluorescence co-localization analysis of desmin and GlcNAc-PEI interaction in fibrotic mice. Scale bar: 200 μm. **(C)** SPECT/CT imaging of control mice and mice at different stages of fibrosis that were injected with ^99m^Tc-GlcNAc-PEI showed the uptake of ^99m^TC-GlcNAc-PEI by different organs. **(D)** The trend of liver uptake of ^99m^Tc-GlcNAc-PEI with disease progression. **(E)** SPECT/CT imaging of ^99m^Tc-GlcNAc-PEI in clodronate liposomes-treated and untreated groups of mice with liver fibrosis. **(F)** hepatic uptake of ^99m^Tc-GlcNAc-PEI in clodronate liposomes-treated and untreated groups of mice with liver fibrosis. Reproduced under terms of the CC-BY license.^[102]^ 2018, The Authors, published by [Ivyspring International Publisher].

**Supplementary Fig. 2**


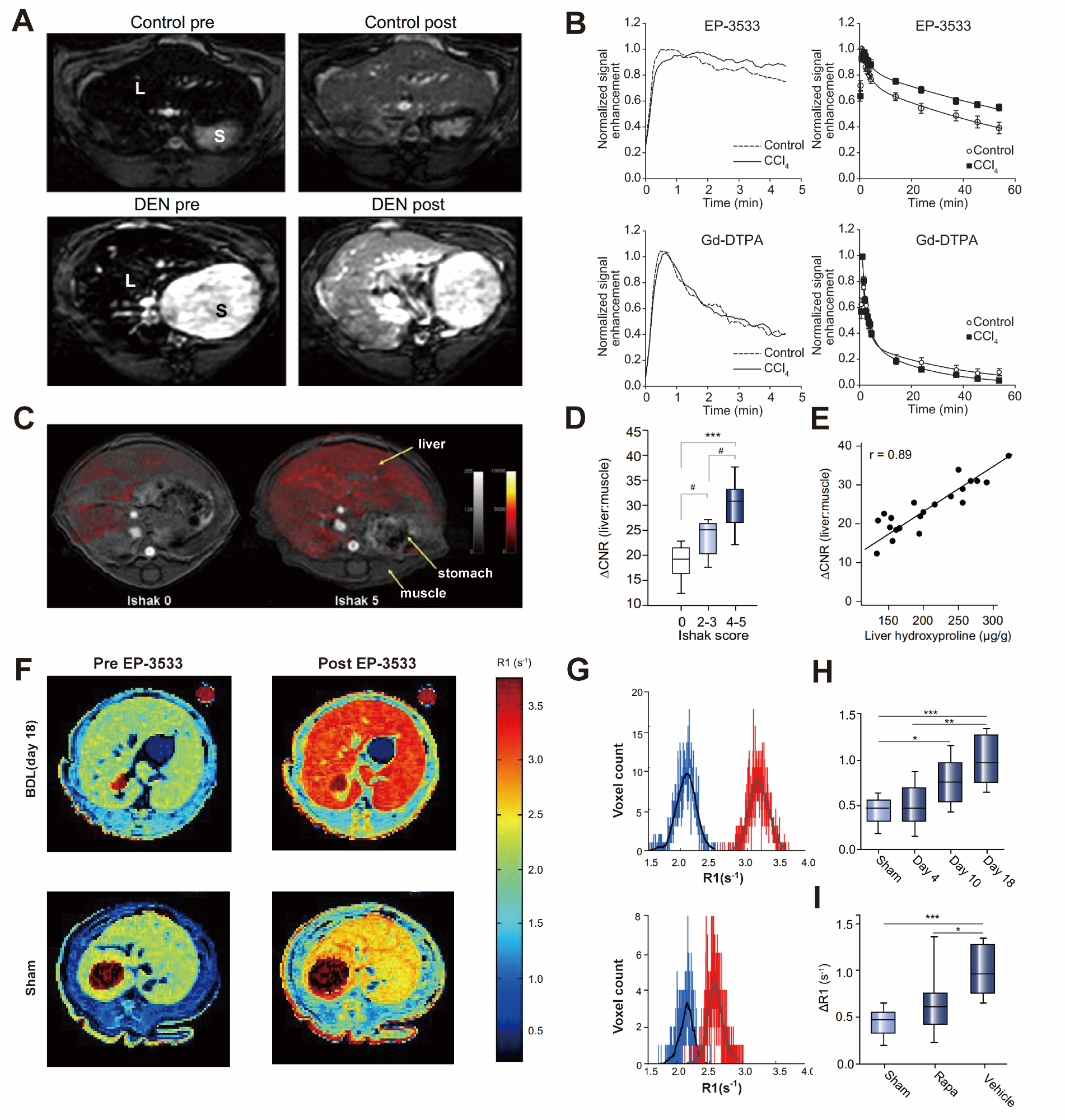


**Supplementary Fig. 2** Molecular targeted imaging of hepatic fibrosis by targeting type I collagen. **(A)** MRI of control and rat DEN model of liver fibrosis before and after injection of EP-3533. **(B)** Plots of MRI in the liver of CCl_4_-treated and control mice before and after injection of EP-3533 and Gd-DTPA. Reproduced with permission.^[13]^ 2012, Elsevier. **(C)** Difference images of axial MRI in control (Ishak 0) and fibrotic (Ishak 5) after and before EP-3533 injection. **(D)** ∆CNR (liver: muscle) of fibrotic CCl_4_-treated mice increased with disease progression (Ishak score) after injection of EP-3533. **(E)** Correlation between ∆CNR (liver: muscle) and total collagen (hydroxyproline). Reproduced with permission.^[128]^ 2013, Elsevier. **(F)** Liver ∆R1 maps obtained before and after EP-3533 in BDL-treated liver fibrosis rats and sham rats. **(G)** Associated histograms of ∆R1 values obtained before (blue) and after EP-3533 (red) in BDL-treated liver fibrosis rats and sham rats. **(H)** ∆R1 gradually increased with time in fibrotic mice after injection of EP-3533 compared to the sham animals. **(I)** Rapamycin-treated mice with liver fibrosis had significantly lower ∆R1 than controls. Reproduced with permission.^[130]^ 2015, Elsevier.**Supplementary Fig. 3**

**
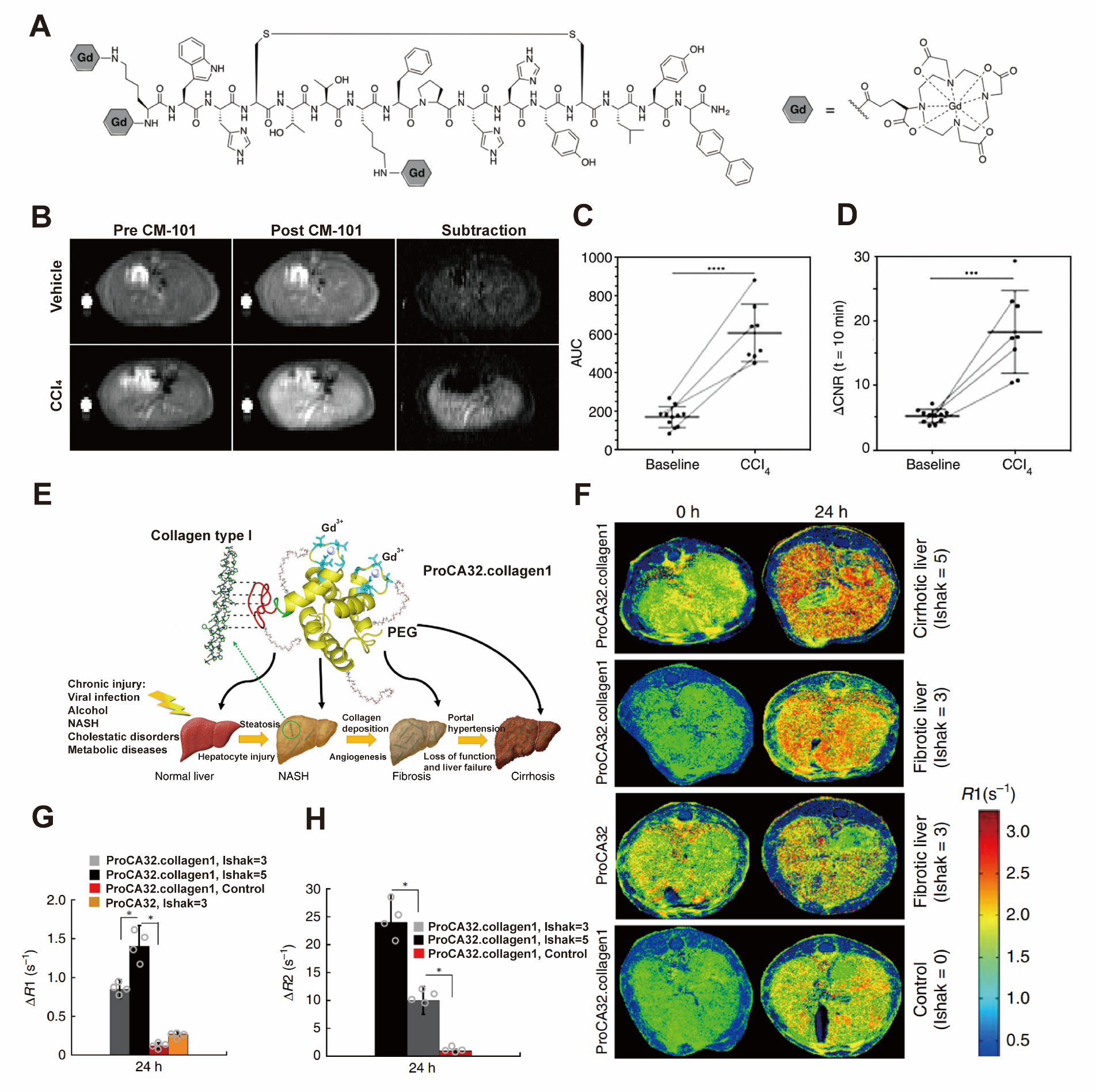
**

**Supplementary Fig. 3** Gd-based contrast agents for MRI of liver fibrosis. **(A)** Schematic structure of CM-101 containing three Gd-DOTA chelates. **(B)** Representative T1-weighted and subtracted images of CCl_4_-treated and control mice obtained before and after CM-101 injection. **(C)** Significant differences in the AUC between baseline and CCl_4_-treated mice after injection of CM-101. **(D)** Significant differences in the ∆CNR between baseline and CCl_4_-treated mice after CM-101 injection. Reproduced with permission.^[143]^ 2018, RSNA. **(E)** Model structure of ProCA32.collagen1 and schematic representation of its binding to type I collagen. **(F)** R1 maps of mice with different liver fibrosis stages and control mice before and after injection of ProCA32.collagen1 (targeted) and ProCA32 (non-targeted). **(G)** ΔR1 values derived from maps of ProCA32.collagenl increased with the progression of liver fibrosis compared to ProCA32. **(H)** ΔR2 values derived from maps of ProCA32.collagenl increased with the progression of liver fibrosis compared to ProCA32. Reproduced under terms of the CC-BY license.^[144]^ 2019, The Authors, published by [Springer Nature].**Supplementary Fig. 4**

**
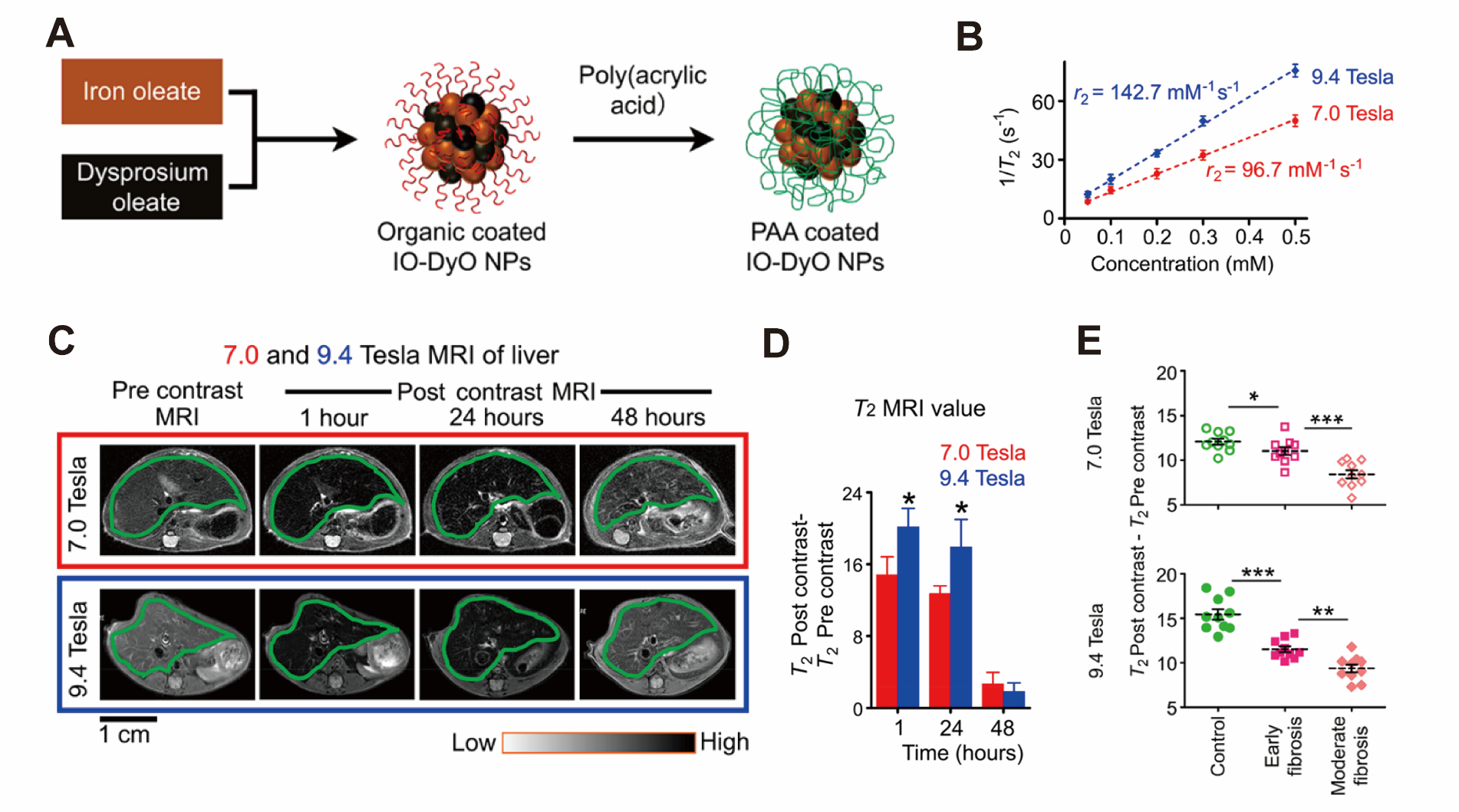
**

**Supplementary Fig. 4** Iron oxide-based for MRI of liver fibrosis. **(A)** Schematic representation of the synthesis of IO-DyO NPs. **(B)** Linear graphs of T2 values measured from different concentrations of IO-DyO NPs solutions at 7.0 and 9.4 T. **(C)** T2-weighted MRI of the liver before and after injection of IO-DyO NPs at 7.0 and 9.4 T in fibrotic mice. **(D)** Values of T2 MRI relaxivity of IO-DyO NPs peaked within 1 h and remained stable for nearly 24 h after injection of IO-DyO NPs at 7.0 and 9.4 T. **(E)** Significant differences in MRI signals between different liver fibrosis stages after *in vivo* injection of IO-DyO NPs at 7.0 and 9.4 T. Reproduced with permission.^[149]^ 2022, American Chemical Society.

**Supplementary Fig. 5**

**
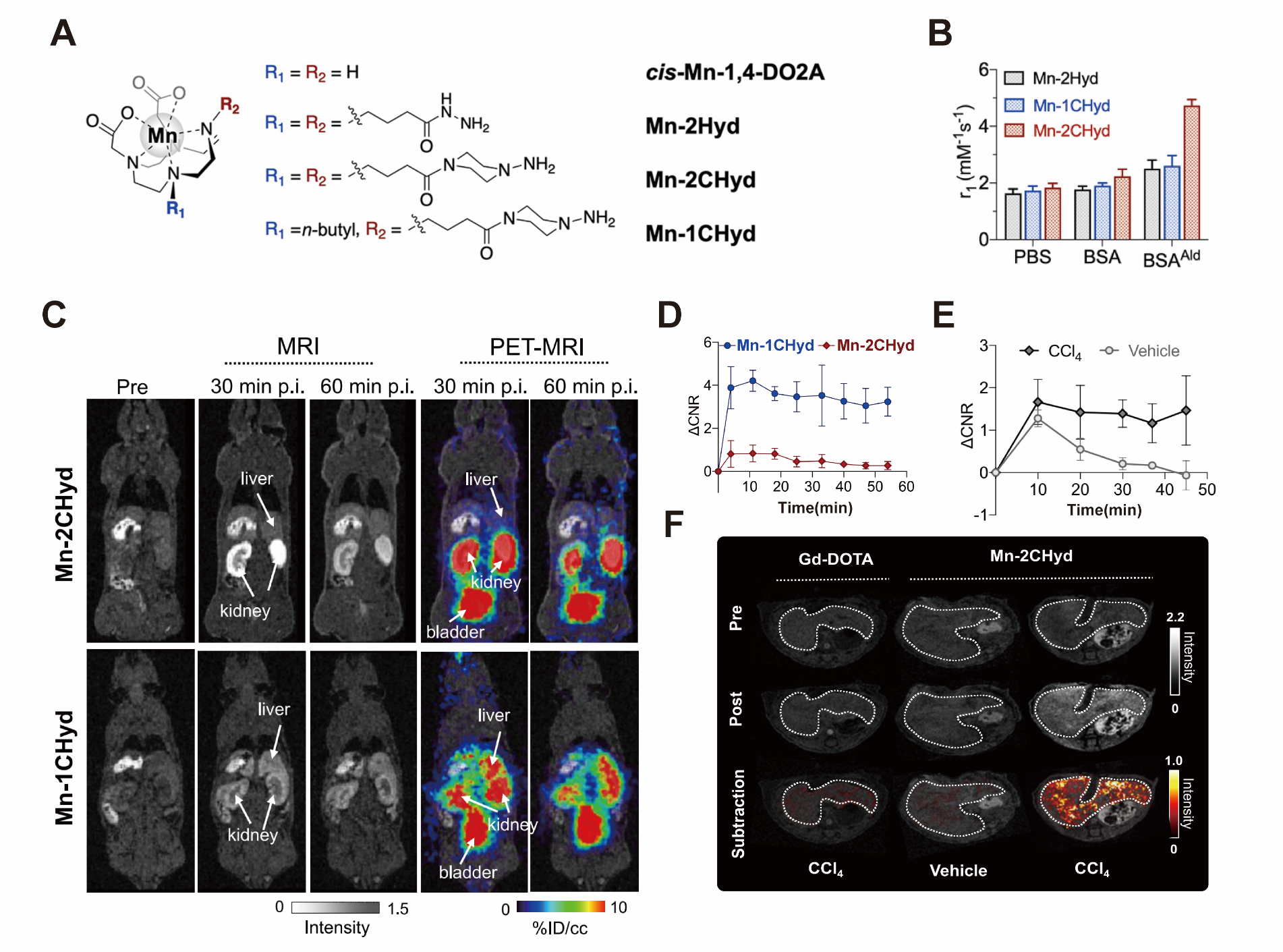
**

**Supplementary Fig. 5** Mn-based contrast agents for MRI of liver fibrosis. **(A)** Schematic chemical structure of Mn-2CHyd, Mn-2Hyd, and Mn-1Chyd (controls). **(B)** Relaxivity values of Mn-2CHyd, Mn-1CHyd, and Mn-2Hyd in PBS alone or with BSA or BSA^Ald^. **(C)** Whole-body MRI and PET-MRI images of normal mice were taken pre-, 30, and 60 mins after injection of [^52^Mn]Mn-2CHyd or [^52^Mn]Mn-1CHyd. **(D)** The change in the liver-to-muscle MRI ΔCNR over time for Mn-2CHyd or Mn-1CHyd in normal mice. **(E)** The post-pre difference in the liver-to-muscle ΔCNR over time for mice treated with CCl_4_ and olive oil vehicle imaged with Mn-2CHyd. **(F)** Liver images of CCl_4_- and olive oil vehicle-treated mice taken before and after injection of Gd-DOTA or Mn-2CHyd, and corresponding subtracted images of liver images. Reproduced with permission.^[156]^ 2022, American Chemical Society.

**Supplementary Fig. 6**

**
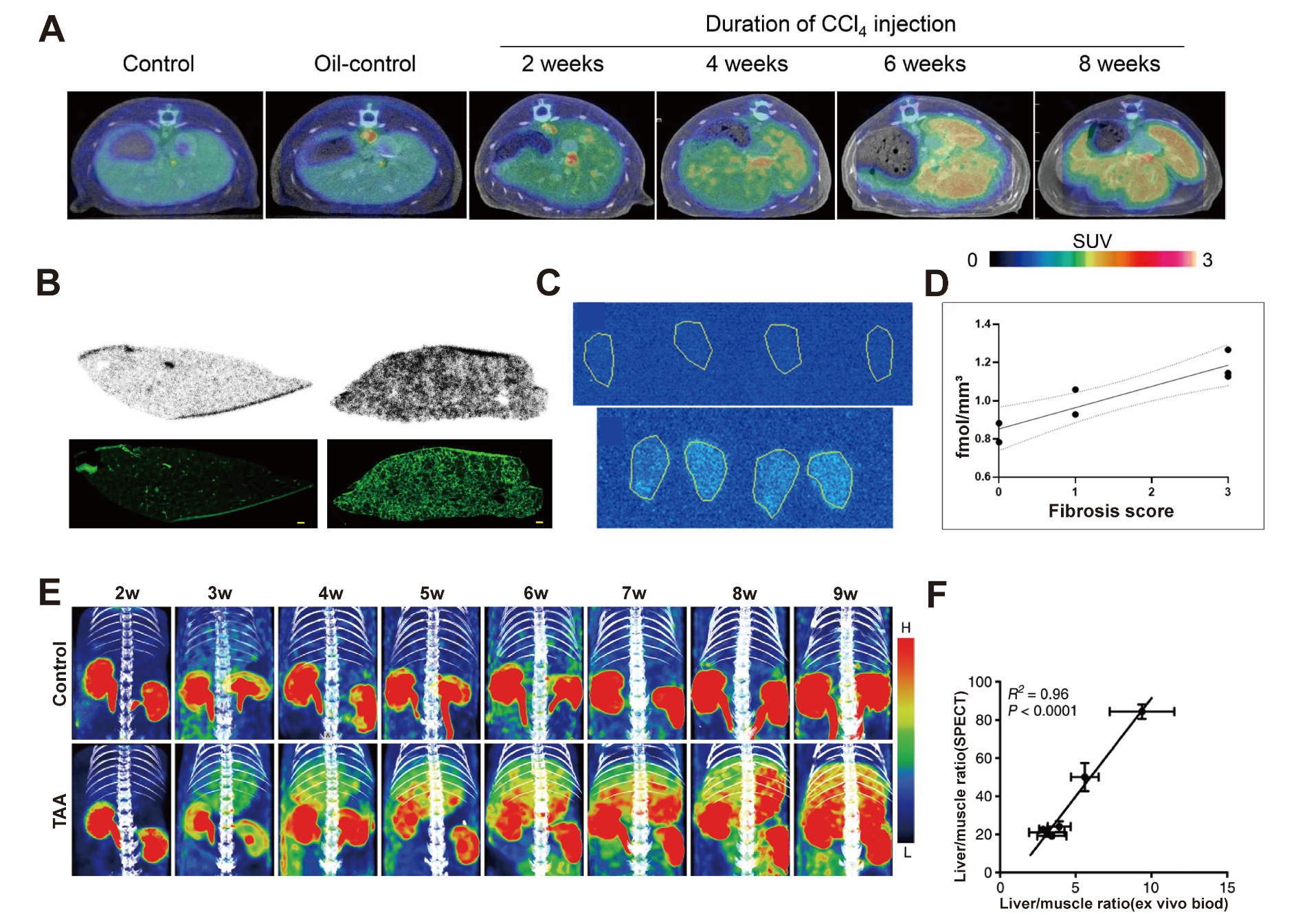
**

**Supplementary Fig. 6** PET and SPECT are used for liver fibrosis. **(A)** Radioactive accumulation of [^18^F]FEDAC in the liver of control and CCl_4_-treated rats for different durations. **(B)** Relationship between [^18^F]FEDAC radioactivity distribution (above) and TSPO expression (below) in the livers of control and CCl_4_-treated rats, respectively. Scale bar: 1 mm. Reproduced under terms of the CC-BY license.^[163]^ 2015, The Authors, published by [Macmillan Publishers Limited]. **(C)** Radiographic autoradiography of frozen sections of ordinary (above) and fibrotic mouse livers (below). **(D)** Correlation of [^68^Ga]Ga-NO2A-[Nle^13^]-Col uptake with a fibrosis score in frozen liver sections from fibrotic mice measured by autoradiography. Reproduced under terms of the CC-BY license.^[170]^ 2021, The Authors. **(E)** SPECT/CT images of TAA different stages of liver fibrosis mice group and control rats after ^99m^Tc-3PRGD2 injection. **(F)** Correlation between SPECT/CT-based quantitative analysis and *in vitro* biodistribution analysis. Reproduced with permission.^[160]^ 2015, RSNA.

**Supplementary Fig. 7**

**
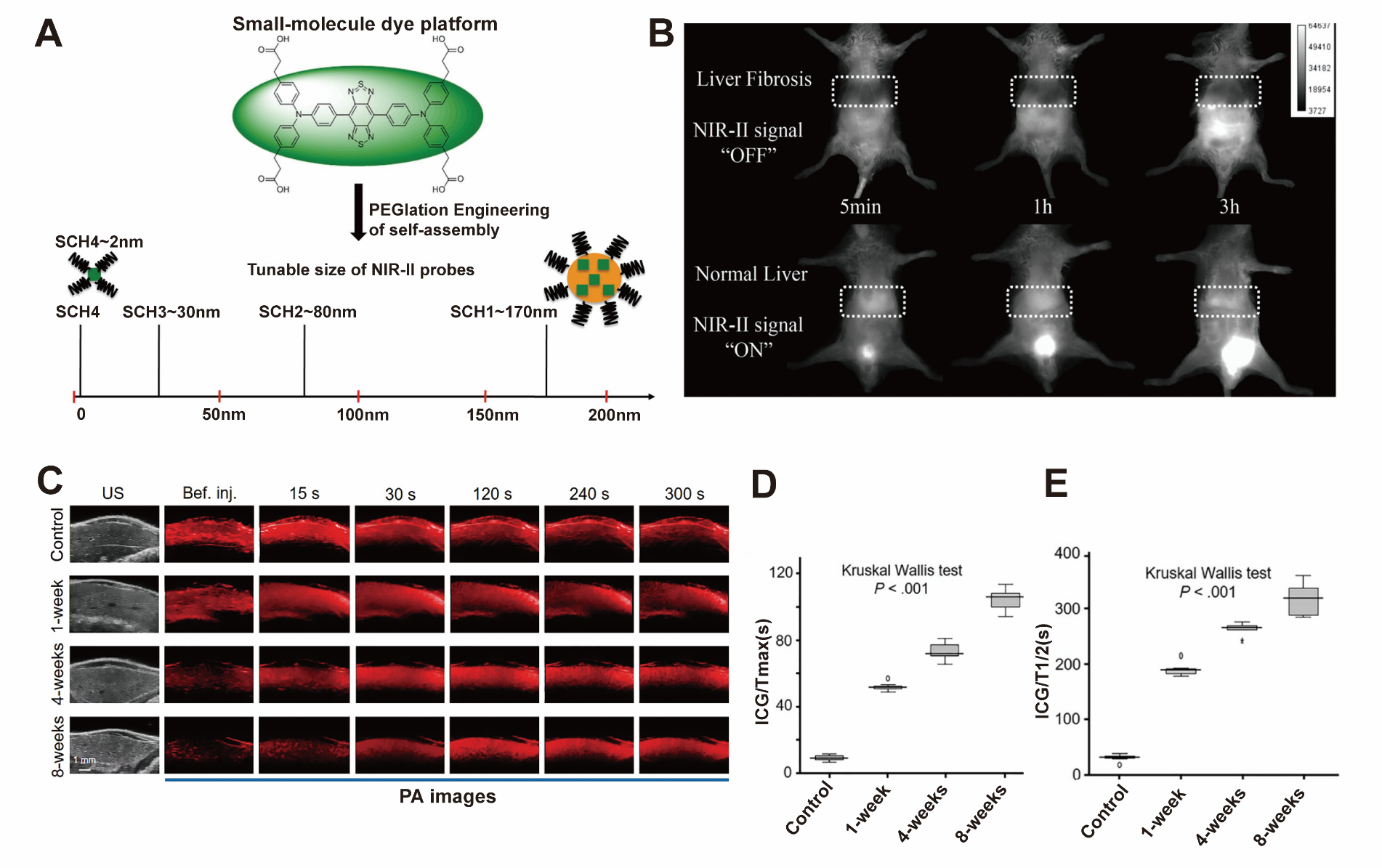
**

**Supplementary Fig. 7** FI and PAI are used for liver fibrosis. **(A)** NIR-II probes (SCH1-SCH4) from single molecule to NPs were assembled based on the PEGylated engineering of CH1055 platform. **(B)** Comparison of NIR-II images of the liver between standard and liver fibrosis groups after SCH4 injection. Reproduced with permission.^[178]^ 2018, John Wiley and Sons. **(C)** Time course of hepatic DCE-PAI in control and fibrotic mice before and after ICG injection. **(D)** Maximum peak time (T_max_) of PAI functional parameters in the liver of hepatic fibrosis mice increased with the fibrosis progression compared to the control group. **(E)** half-life (T_1/2_) of PAI functional parameters in the liver of hepatic fibrosis mice increased with the fibrosis progression compared to the control group. Reproduced with permission.^[183]^ 2021, RSNA.

**Supplementary Fig. 8**


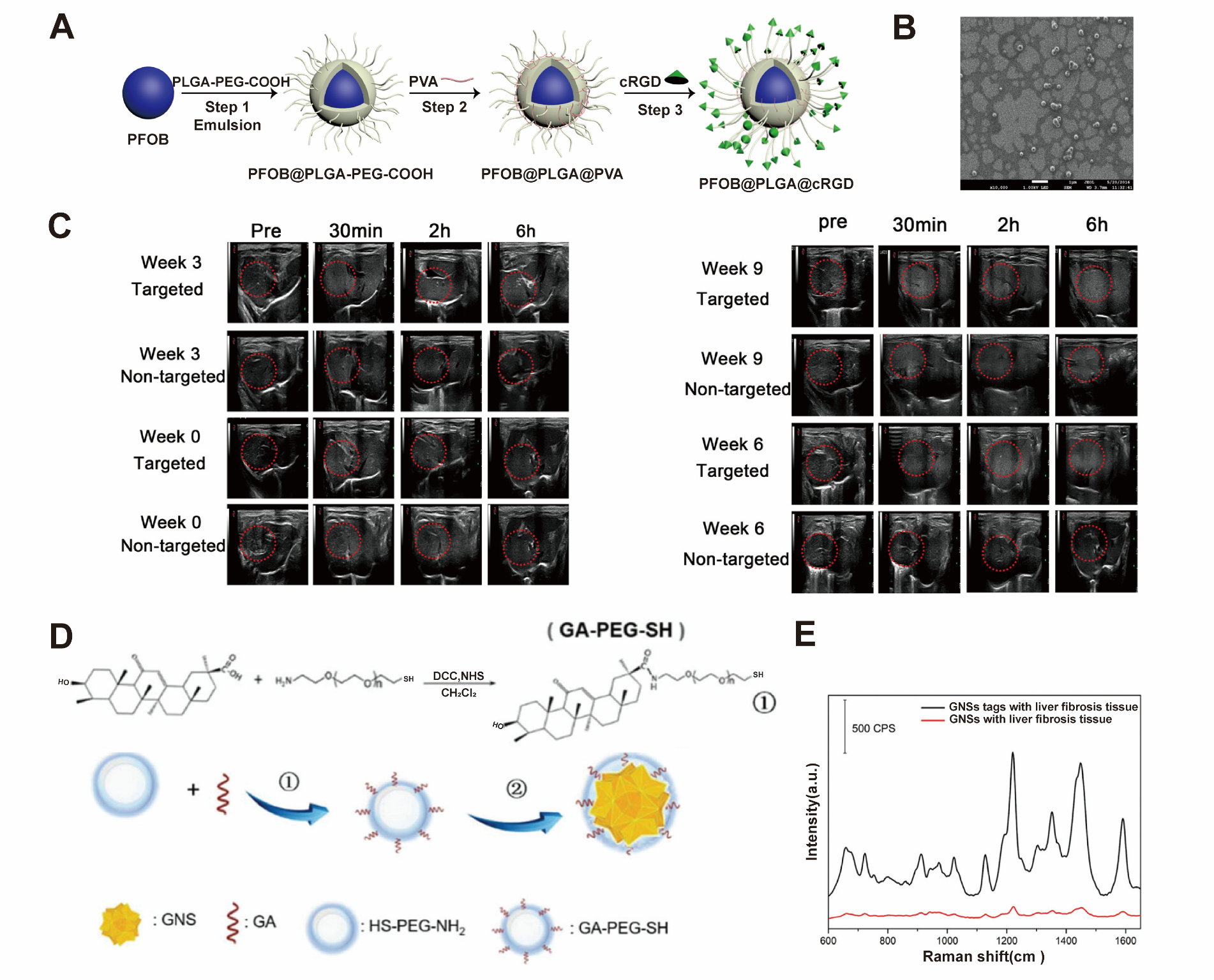


**Supplementary Fig. 8** US and SERS are used for liver fibrosis. **(A)** Schematic structure of cRGD-PLGA-PFOB NPs for targeted molecular imaging. **(B)** SEM image of PLGA-PFOB NPs. Scale, 1 μm. **(C)** Contrast-enhanced liver US images after injection of targeted contrast agents (cRGD-PLGA-PFOB NPs) or non-targeted contrast agents (PLGA-PFOB NPs) in mice with different liver fibrosis periods. Reproduced under terms of the CC-BY license.^[182]^ 2017, The Authors. **(D)** The flow chart of GLTTs preparation. **(E)** The SERS spectra of liver fibrosis issues with GNSs and GLTTs. Reproduced with permission.^[184]^ 2021, The Royal Society of Chemistry.
